# Supplementary material for: Long non-coding RNA PCAT19 safeguards DNA in quiescent endothelial cells by preventing uncontrolled phosphorylation of RPA2
Source: Cell Rep. 2022 Nov 15;41(7):111670. doi: 10.1016/j.celrep.2022.111670 (PMC9681662; doi:10.1016/j.celrep.2022.111670)
Supplement: Document S1. Figures S1–S3 and Table S3 [file mmc1.pdf]

Cell Reports, Volume *41*

Supplemental information

Long non-coding RNA *PCAT19* safeguards DNA in quiescent endothelial cells by preventing uncontrolled phosphorylation of RPA2

James A. Oo, Katalin Pálfi, Timothy Warwick, Ilka Wittig, Cristian Prieto-Garcia, Vigor Matkovic, Ines Tomašković, Frederike Boos, Judit Izquierdo Ponce, Tom Teichmann, Kirill Petriukov, Shaza Haydar, Lars Maegdefessel, Zhiyuan Wu, Minh Duc Pham, Jaya Krishnan, Andrew H. Baker, Stefan Günther, Helle D. Ulrich, Ivan Dikic, Matthias S. Leisegang, and Ralf P. Brandes

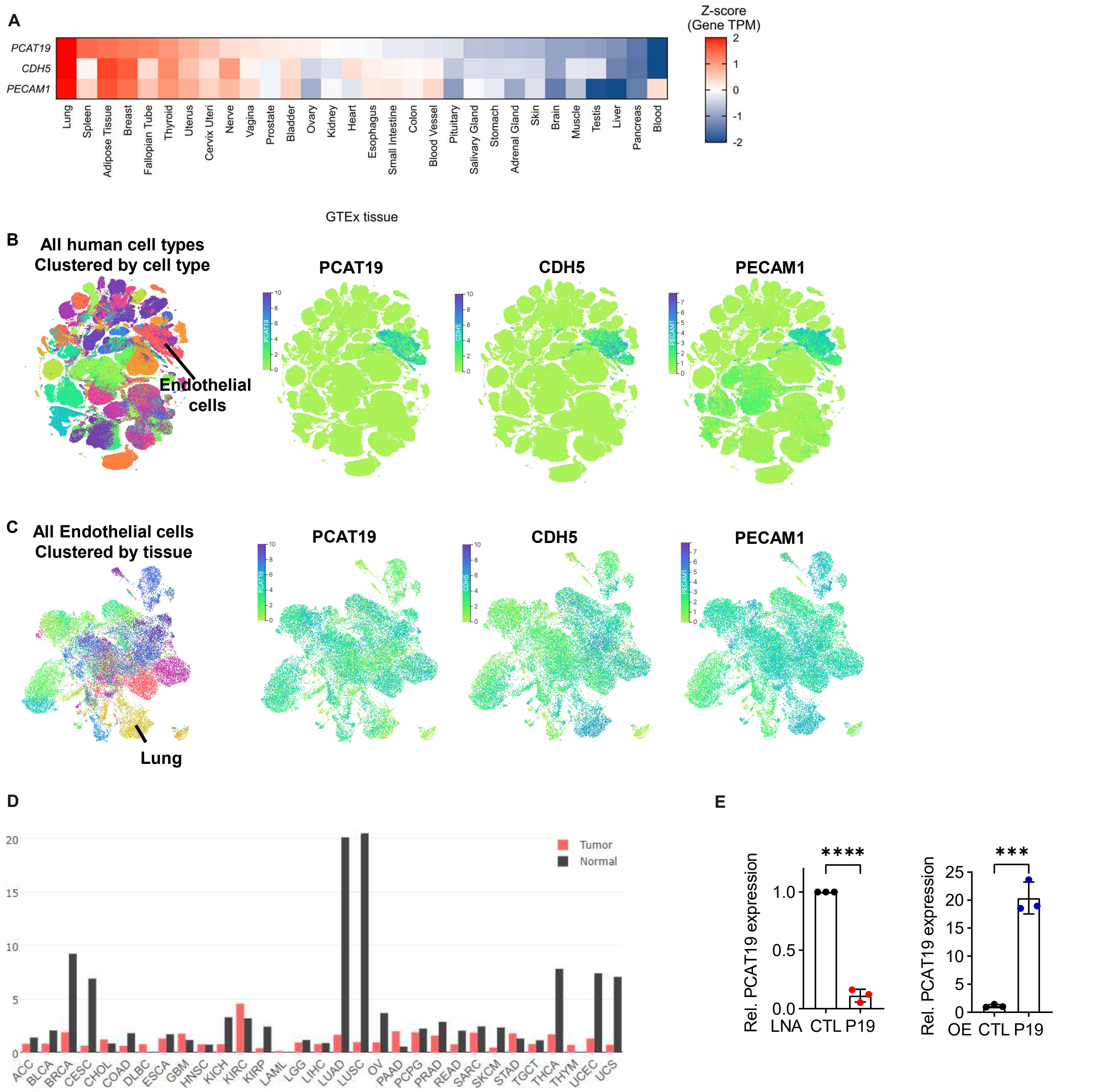

Figure S1. PCAT19 expression, Related to Figures 1 and 2. A. *PCAT19, CDH5* and *PECAM1* expression (Z-score of gene TPM) in normal human tissues from The GTEx Portal (GTEx Analysis Release V8 (dbGaP Accession phs000424.v8.p2). TPM, transcripts per million. **B.** *PCAT19*, *CDH5* and *PECAM1* expression in different human cell types. Clustered by cell type. Tabula Sapiens. **C.** *PCAT19*, *CDH5* and *PECAM1* expression in individual endothelial cells across all human tissues. Clustered by tissue. Tabula Sapiens. **D.** *PCAT19* expression in various tumour tissues and respective normal tissues, GEPIA database. **E.** RT-qPCR for PCAT19 after PCAT19 or control LNA GapmeR-mediated knockdown and PCAT19 or pcDNA3.1+ control overexpression. HUVEC (n=3 biological replicates) for both. Unpaired t-test, *** signifies P<0.001, **** signifies P<0.0001.

A

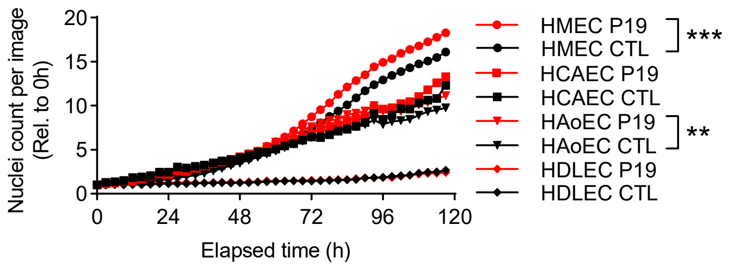

B

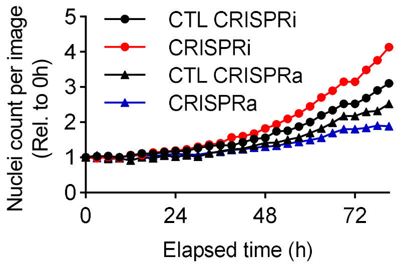

C

ID	Description	GeneRatio	BgRatio	pvalue	p.adjust	Count	FoldEnrichment
hsa04110	Cell cycle	10/78	126/8114	2.9351E-07	4.8135E-05	10	8.25600326
hsa04218	Cellular senescence	9/78	156/8114	1.6739E-05	0.00137261	9	6.00147929
hsa04914	Progesterone-mediated oocyte maturation	7/78	102/8114	5.1302E-05	0.00280453	7	7.13901458
hsa05166	Human T-cell leukemia virus 1 infection	9/78	222/8114	0.00025833	0.01059171	9	4.21725572
hsa04114	Oocyte meiosis	6/78	131/8114	0.0015673	0.05140744	6	4.76453318
hsa04010	MAPK signaling pathway	9/78	294/8114	0.001933	0.05283537	9	3.1844584
hsa05202	Transcriptional misregulation in cancer	7/78	193/8114	0.00245286	0.05746707	7	3.77295071
hsa04115	p53 signaling pathway	4/78	73/8114	0.00520871	0.10677856	4	5.70003512
hsa04657	IL-17 signaling pathway	4/78	94/8114	0.01254742	0.20036465	4	4.42662302
hsa05169	Epstein-Barr virus infection	6/78	202/8114	0.01284993	0.20036465	6	3.08987053
hsa05203	Viral carcinogenesis	6/78	204/8114	0.01343909	0.20036465	6	3.05957768
hsa04933	AGE-RAGE signaling pathway in diabetic complications	4/78	100/8114	0.01545506	0.21121909	4	4.16102564
hsa05161	Hepatitis B	5/78	162/8114	0.01955799	0.24563435	5	3.21066793
hsa04668	TNF signaling pathway	4/78	112/8114	0.02246656	0.24563435	4	3.71520147
hsa05145	Toxoplasmosis	4/78	112/8114	0.02246656	0.24563435	4	3.71520147
hsa04935	Growth hormone synthesis, secretion and action	4/78	119/8114	0.02732572	0.28008868	4	3.4966602
hsa04917	Prolactin signaling pathway	3/78	70/8114	0.02952985	0.28487624	3	4.45824176
hsa04380	Osteoclast differentiation	4/78	128/8114	0.03443394	0.29341409	4	3.25080128
hsa04926	Relaxin signaling pathway	4/78	129/8114	0.03528429	0.29341409	4	3.22560127
hsa04068	FoxO signaling pathway	4/78	131/8114	0.03702153	0.29341409	4	3.17635545
hsa04215	Apoptosis - multiple species	2/78	32/8114	0.03757132	0.29341409	2	6.50160256
Hsa05162	Measles	4/78	139/8114	0.04445916	0.31701313	4	2.99354363
Hsa05418	Fluid shear stress and atherosclerosis	4/78	139/8114	0.04445916	0.31701313	4	2.99354363

D

KEGG Description	P-adjusted Value	Gene count	Genes
Cell cycle	4.824335e-05	10	*BUB1, BUB1B, CCNA2, CCNB2, CCND2, CDC20, CDC25B, ESPL1, ORC1, TTK*
Cellular senescence	1.375276e-03	9	*CCNA2, CCNB2, CCND2, FOXM1, IGFBP3, MAP2K6, MAPK14, MYBL2, TGFBR2*
Progesterone-mediated oocyte maturation	2.808863e-03	7	*BUB1, CCNA2, CCNB2, CDC25B, CPEB4, KIF22, MAPK14*
Human T-cell leukemia virus 1 infection	1.061092e-02	9	*BUB1B, CCNA2, CCNB2, CCND2, CDC20, ESPL1, NFKB2, STAT5A, TGFBR2*

E

Symbol	Regulation	Significant reachable	*P*-value
AURKB	down	12	2.42E-10
PLK1	down	10	4.41E-09
CDK1	down	12	1.50E-07
SMARCA2	up	11	6.42E-05
SMARCA4	up	11	7.12E-05
RB1	down	6	7.32E-05
E2F3	down	7	0.000304
CCNB1	down	10	0.000319

Figure S2. PCAT19 on endothelial cell cycle, Related to Figure 2. A. Proliferation measured with Incucyte imaging system after LNA GapmeR-mediated knockdown of PCAT19 (P19) compared to negative control (CTL) LNA GapmeR for HMEC, HCAEC, HAoEC and HDLEC. n=2 technical replicates, unpaired t-test of Area under the curve (AUC). ** signifies $P<0.01$, *** signifies $P<0.001$. **B.** Endothelial cell proliferation measured with Incucyte imaging system after PCAT19 CRISPRi or CRISPRa or respective negative controls. n=3 biological replicates. **C.** KEGG (Kyoto Encyclopedia of Genes and Genomes) pathway enrichment list from 186 differentially regulated genes after *PCAT19* knockdown. **D.** Differentially expressed genes associated with the top significant KEGG pathways (*P*adj<0.05). **E.** Upstream regulator analysis (QuaternaryProd package). Top predicted regulators listed according to their number of significant downstream targets. ** signifies $P<0.01$, *** signifies $P<0.001$.

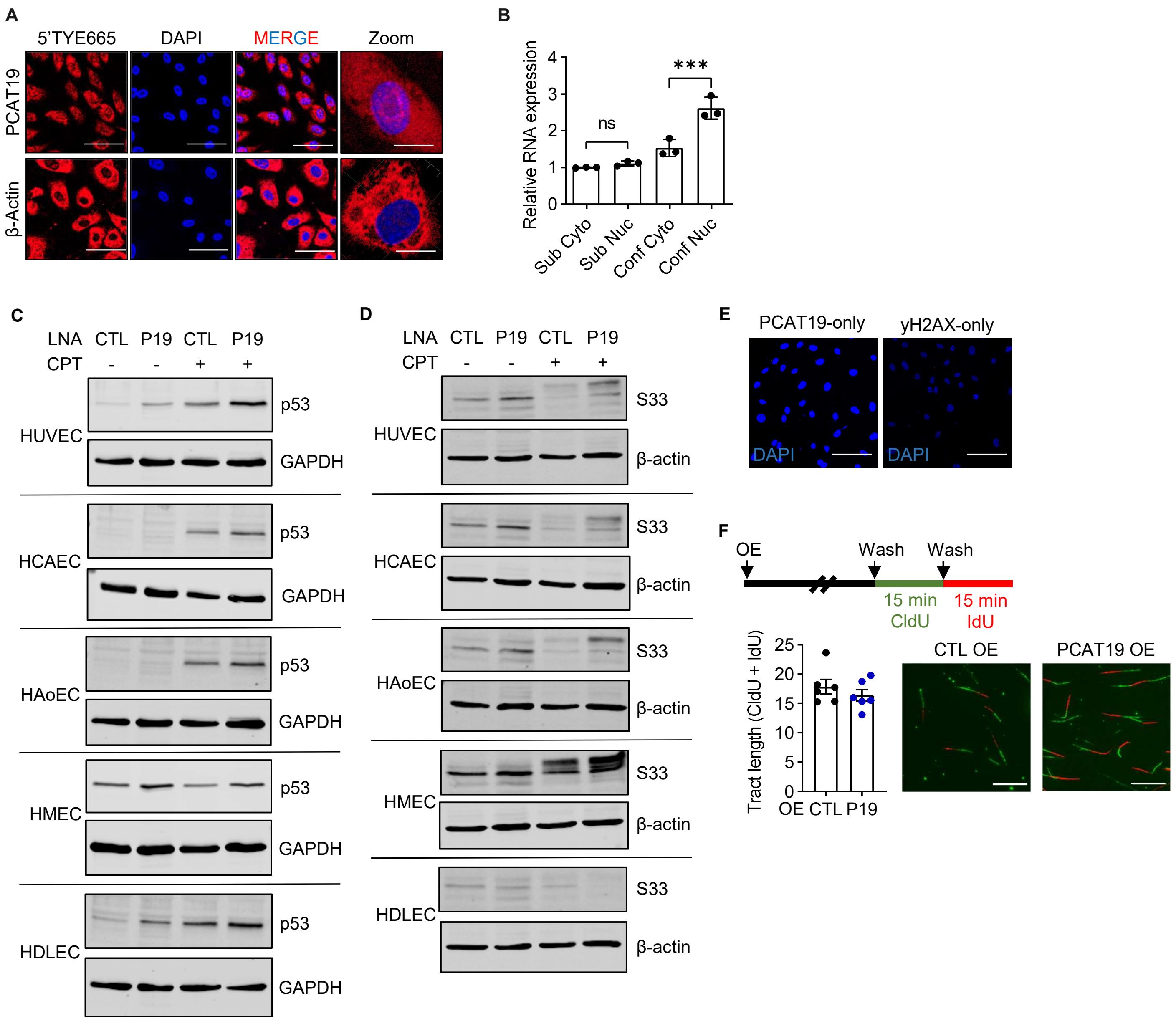

Figure S3. PCAT19 subcellular localisation and effects on DNA damage markers and DNA replication, Related to Figures 1 and 4. A. RNA-FISH for *PCAT19* and β-Actin localisation in HUVEC. Respective antisense-oligonucleotide probes tagged with 5'TYE665 (647nm). DAPI counterstaining. Scale bar represents 100 µm and "Zoom" image scale bars represent 25 µm. **B.** HUVEC fractionation into cytoplasm (Cyto) and nucleus (Nuc) after cell growth under both subconfluent (sub) and confluent (conf) conditions. RNA isolation and RT-qPCR for *PCAT19* and GAPDH. n=3, unpaired t-test, mean ± SD. **C.** HUVEC, HCAEC, HaoEC, HMEC and HDLEC were transfected with PCAT19 LNA (P19) or negative control LNA (CTL) and then treated with DMSO or camptothecin (CPT). n=1. Western blot staining for p53 and GAPDH or **D.** S33-pRPA2 (S33) and β-actin. **E.** RNA In Situ Hybridization Proximity Ligation Assay (rISH-PLA) PCAT19- and γH2AX-only controls. Red signal indicates PLA signal (546nm) between PCAT19 and γH2AX, blue indicates DAPI. Scale bar represents 100 µm **F.** HUVEC were transduced with PCAT19 or pcDNA3.1+ control plasmids and pulsed with CldU and IdU for the DNA fibre assay. Quantification of fibres and representative images are displayed. (n=6) Scale bar represents 15 µm. *** signifies *P*<0.001.

Table S3. Primers used in this study, Related to Figures 2 and 3.

Name	Sequence (5'-3')
18S rRNA FP	CTT TGG TCG CTC GCT CCT C
18S rRNA RP	CTG ACC GGG TTG GTT TTG AT
GAPDH FP	TGC ACC ACC AAC TGC TTA GC
GAPDH RP	GGC ATG GAC TGT GGT CAT GAG
PCAT19 FP	ACC CTG CCC TTA GTC AAA TC
PCAT19 RP	TGG AAT CCC ACA CTG TTA CC
U4 snRNA FP	GCC AAT GAG GTT TAT CCG AGG
U4 snRNA RP	TCA AAA ATT GCC AAT GCC G
