# Supplementary material for: Long non-coding RNA PCAT19 safeguards DNA in quiescent endothelial cells by preventing uncontrolled phosphorylation of RPA2
Source: Cell Rep. 2022 Nov 15;41(7):111670. doi: 10.1016/j.celrep.2022.111670 (PMC9681662; doi:10.1016/j.celrep.2022.111670)
Supplement: Document S2. Article plus supplemental information [file mmc4.pdf]

Cell Reports

Article

Long non-coding RNA *PCAT19* safeguards DNA in quiescent endothelial cells by preventing uncontrolled phosphorylation of RPA2

Graphical abstract

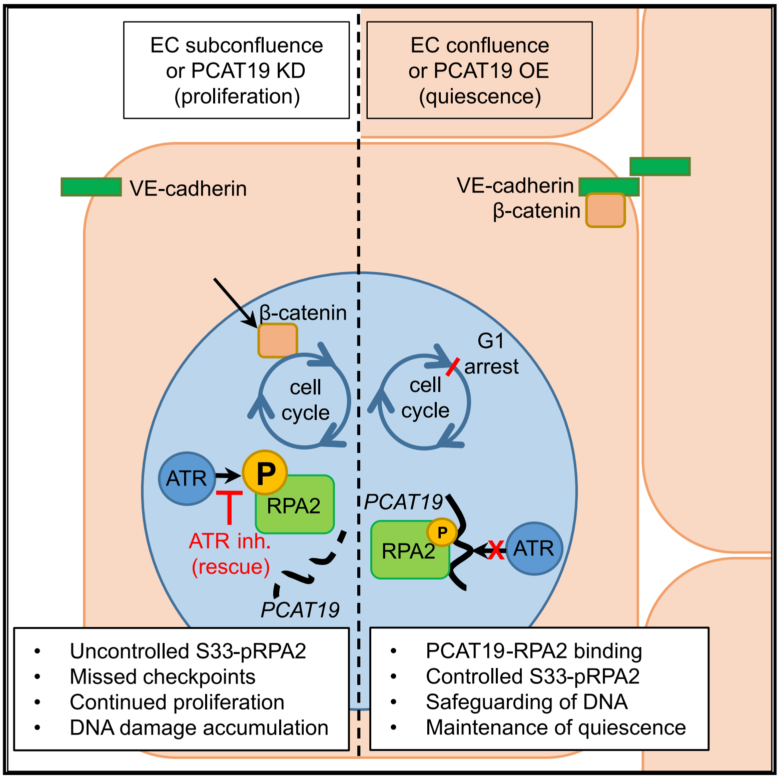

Authors

James A. Oo, Katalin Pálfi,
Timothy Warwick, ..., Ivan Dikic,
Matthias S. Leisegang, Ralf P. Brandes

Correspondence

leisegang@vrc.uni-frankfurt.de (M.S.L.),
brandes@vrc.uni-frankfurt.de (R.P.B.)

In brief

Oo et al. report an essential role for the lncRNA *PCAT19* in the endothelial cell proliferation-quiescence switch. Endothelial cell-enriched *PCAT19* is upregulated with quiescence and binds RPA2 to prevent aberrant phosphorylation of RPA2-S33 by ATR kinase. This ensures an appropriate DNA damage response for the endothelial cell-cycle state.

Highlights

- Endothelial cell-enriched lncRNA *PCAT19* is upregulated with quiescence
- *PCAT19* depletion promotes cell proliferation, sprouting, and DNA damage
- *PCAT19* binds to RPA2 and blocks RPA2-S33 phosphorylation by the ATR kinase
- Blocking ATR activity rescues the endothelial phenotype after *PCAT19* depletion

Oo et al., 2022, Cell Reports *41*, 111670
November 15, 2022 © 2022 The Author(s).
https://doi.org/10.1016/j.celrep.2022.111670

CellPress

Cell Reports

CellPress
OPEN ACCESS

Article

Long non-coding RNA *PCAT19* safeguards DNA in quiescent endothelial cells by preventing uncontrolled phosphorylation of RPA2

James A. Oo,[1,6] Katalin Pálfi,[1,6] Timothy Warwick,[1,6] Ilka Wittig,[1,6,7] Cristian Prieto-Garcia,[3] Vigor Matkovic,[3,14] Ines Tomašković,[3] Frederike Boos,[1,6] Judit Izquierdo Ponce,[1] Tom Teichmann,[1,6] Kirill Petriukov,[4] Shaza Haydar,[1,6] Lars Maegdefessel,[8,10] Zhiyuan Wu,[8,10] Minh Duc Pham,[9,11] Jaya Krishnan,[6,9,12] Andrew H. Baker,[5,13] Stefan Günther,[2] Helle D. Ulrich,[4] Ivan Dikic,[3,14,15] Matthias S. Leisegang,[1,6,*] and Ralf P. Brandes[1,6,16,*]

[1]Institute for Cardiovascular Physiology, Goethe University, Theodor-Stern-Kai 7, 60596 Frankfurt, Germany
[2]Max Planck Institute for Heart and Lung Research, 61231 Bad Nauheim, Germany
[3]Institute of Biochemistry II, Faculty of Medicine, Goethe University, 60596 Frankfurt, Germany
[4]Institute of Molecular Biology (IMB), 55128 Mainz, Germany
[5]The Queen's Medical Research Institute, Centre for Cardiovascular Science, University of Edinburgh, Edinburgh EH16 4TJ, Scotland
[6]German Center of Cardiovascular Research (DZHK), Partner Site RheinMain, Frankfurt, Germany
[7]Functional Proteomics, Institute for Cardiovascular Physiology, Goethe University, 60596 Frankfurt, Germany
[8]Department of Vascular and Endovascular Surgery, Klinikum rechts der Isar-Technical University Munich, 81675 Munich, Germany
[9]Institute of Cardiovascular Regeneration, Center for Molecular Medicine, Goethe University, 60596 Frankfurt, Germany
[10]German Center of Cardiovascular Research (DZHK), Partner Site Munich, Munich, Germany
[11]Genome Biologics, Theodor-Stern-Kai 7, 60596 Frankfurt, Germany
[12]Cardio-Pulmonary Institute, Giessen, Germany
[13]CARIM Institute, University of Maastricht, Universiteitssingel 50, 6200 Maastricht, the Netherlands
[14]Buchmann Institute for Molecular Life Sciences, Goethe University, 60438 Frankfurt, Germany
[15]Max Planck Institute of Biophysics, Max-von-Laue Straße 3, 60438 Frankfurt, Germany
[16]Lead contact
*Correspondence: leisegang@vrc.uni-frankfurt.de (M.S.L.), brandes@vrc.uni-frankfurt.de (R.P.B.)
https://doi.org/10.1016/j.celrep.2022.111670

SUMMARY

In healthy vessels, endothelial cells maintain a stable, differentiated, and growth-arrested phenotype for years. Upon injury, a rapid phenotypic switch facilitates proliferation to restore tissue perfusion. Here we report the identification of the endothelial cell-enriched long non-coding RNA (lncRNA) *PCAT19*, which contributes to the proliferative switch and acts as a safeguard for the endothelial genome. *PCAT19* is enriched in confluent, quiescent endothelial cells and binds to the full replication protein A (RPA) complex in a DNA damage- and cell-cycle-related manner. Our results suggest that *PCAT19* limits the phosphorylation of RPA2, primarily on the serine 33 (S33) residue, and thereby facilitates an appropriate DNA damage response while slowing cell cycle progression. Reduction in *PCAT19* levels in response to either loss of cell contacts or knockdown promotes endothelial proliferation and angiogenesis. Collectively, *PCAT19* acts as a dynamic guardian of the endothelial genome and facilitates rapid switching from quiescence to proliferation.

INTRODUCTION

Endothelial cells (ECs) form the innermost layer of blood vessels and are indispensable for vascular patterning and homeostasis. This patterning is required for vascular development and includes sprouting and branching, with the density of the vascular network being further adjusted by vessel regression.[10,11] To maintain a functional monolayer, endothelial cells must switch from a proliferative to a quiescent state while remaining primed for re-entry into the cell cycle.[12] Contact inhibition and quiescence of the cell cycle are triggered by the contact of cell-to-cell junctions, through VE-cadherin clustering, in particular.[13–15] VE-cadherin is a transmembrane protein linked to p120-catenin and β-catenin, which are retained with VE-cadherin in the cytoplasm under confluent conditions, thereby preventing their transcriptional activity at genes involved in cell cycle progression. VE-cadherin also interacts with VEGFR2 to prevent its proliferative signaling.[13] Ultimately, multiple signaling pathways converge to halt the cell cycle in a controlled and coordinated fashion upon endothelial cell monolayer confluence. Conversely, upon vascular injury or loss of contact inhibition due to vessel outgrowth, the endothelial cell cycle is rapidly reinstated. In addition to cell cycle control in response to environmental cues, extensive intrinsic cell cycle mechanisms have evolved to coordinate, safeguard, and potentially correct the individual steps of the cell cycle.[16]

© 2022 The Author(s).
This is an open access article under the CC BY license (http://creativecommons.org/licenses/by/4.0/).

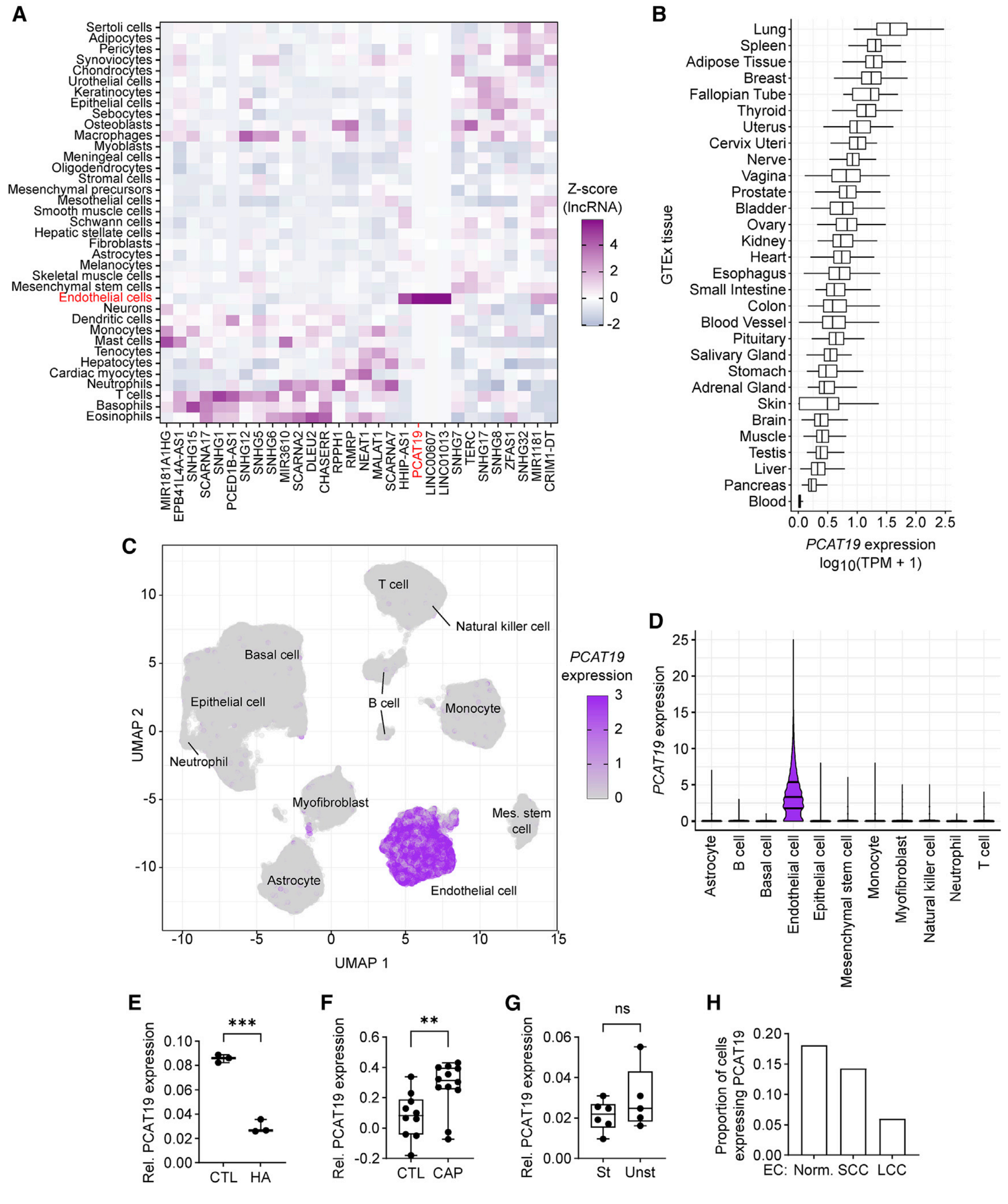

Figure 1. *PCAT19* is highly enriched in endothelial cells and is differentially expressed in vascular diseases

(A) FANTOM5 CAGE expression of the 30 most highly expressed endothelial lncRNAs across different cell types. The *Z* score across cell types for each lncRNA is shown.

(B) *PCAT19* expression ($\log_{10}$(TPM + 1)) in normal human tissues from the GTEx portal (GTEx Analysis Release v.8; dbGaP accession phs000424.v8.p2). TPM, transcripts per million.

(C and D) Uniform manifold approximation and projection (UMAP) plot (C) and violin plot (D) of published scRNA-seq from healthy prostate tissue.[5] Cell types and respective normalized *PCAT19* expression are displayed.

(E) *PCAT19* expression (relative fragments per kilobase of transcript per million mapped reads) in healthy vessel (CTL) and hemangioma (HA). *PCAT19* expression was normalized to PECAM1 expression.

(F) *PCAT19* expression relative to PECAM1 expression in healthy/early carotid artery plaque vessel samples (CTL) (n = 10) or advanced carotid artery plaque (CAP) samples (n = 12) from the Munich Vascular Biobank (30781475).

(legend continued on next page)

A central regulator of the genome maintenance machinery is the single-stranded DNA (ssDNA)-binding replication protein A (RPA) complex, which acts during the initiation and elongation steps of DNA replication and during DNA damage.[17] The complex consists of RPA1, RPA2, and RPA3. Of these, RPA2 is the most important with regard to RPA regulation, as it is heavily controlled by post-translational modifications, particularly phosphorylation.[18] RPA2 is sequentially phosphorylated by three phosphoinositide 3-kinase (PI3K)-like protein kinases (ATR, ATM, and DNA-PK) in response to varying degrees of DNA damage. Phosphorylation of the serine 33 (S33) residue by ATR occurs during S phase in response to replicative stress while signaling the progression of the cell cycle.[19,20] If DNA damage is extensive, subsequent hyperphosphorylation of RPA2 is mediated by ATM and DNA-PK, particularly at the S4/8 residue.[21] This triggers the cell cycle checkpoints and the DNA damage response. Following S33 phosphorylation by ATR, RPA2 can also be phosphorylated at its two cyclin-CDK sites by cyclin B-Cdk1 during mitosis[22] and by cyclin A-Cdk2 at the G1/S boundary.[23]

RPA is involved in multiple DNA-repair pathways, such as nucleotide excision repair (NER), base excision repair (BER), mismatch repair (MMR), and homologous recombination (HR). Mutations in RPA are known to cause DNA damage accumulation due to faulty G1, S, and G2/M checkpoint signaling, which is in part a consequence of insufficient loading of the ATR kinase onto DNA.[18] ATR is normally activated on RPA-coated ssDNA to activate proteins such as Chk1, p53, and downstream cyclins to trigger cell cycle arrest and promote DNA repair. As such, problems with RPA activation and loading onto ssDNA disrupt ATR signaling and predispose the cell to faulty checkpoint signaling and genome instability. Importantly, hyperphosphorylation of free RPA2 not bound to DNA hinders its subsequent loading onto DNA and thereby reduces the effectiveness of the DNA damage response.[22,24] While the main proteins involved in this fundamental pathway have been characterized, a growing body of evidence suggests that RNAs, and in particular long non-coding RNAs (lncRNAs), act on the cell cycle and contribute to cellular proliferation, the DNA damage response, and the maintenance of DNA integrity.[25–27]

lncRNAs are RNAs longer than 200 nt that do not have an apparent protein-coding potential.[28] They are now believed to contribute to numerous cellular processes both within and outside of the nucleus. In the nucleus, lncRNAs can control processes such as transcription, chromatin organization, and the maintenance of genome integrity.[29] With respect to the RPA complex, a recent study identified the lncRNA Discn as being crucial for the regulation of RPA availability in stem cells.[30] Discn is induced under genotoxic stress to prevent the nuclear translocation of nucleolin, a protein that sequesters RPA, thereby preventing RPA exhaustion. The lncRNA telomeric-repeat-containing RNA (TERRA) prevents the displacement of RPA from telomeric ssDNA during the early-to-middle S phase by sequestering heterogeneous nuclear ribonucleoproteins (hnRNPs).[31] When TERRA expression declines toward the end of S phase, hnRNPs displace RPA from ssDNA to reduce ATR activation and allow ssDNA coating by protection of telomeres 1 (POT1) until the next round of DNA replication. This highlights a tightly controlled cell-cycle-dependent function of RPA that is mediated through the expression of a single lncRNA. Given the great importance of the cell cycle and DNA damage response and considering that the human genome codes for more than 30,000 lncRNAs, it is evident that the lncRNAs characterized so far represent only the tip of the iceberg.

Here we set out to uncover endothelial-enriched lncRNAs that play a role in cell cycle regulation and angiogenesis and which therefore might offer a therapeutic target in vascular disease. This led to the identification of the lncRNA prostate cancer-associated transcript 19 (*PCAT19*), which is highly enriched in the confluent endothelium. Our study revealed that *PCAT19* is induced by endothelial quiescence to protect RPA2 from uncontrolled phosphorylation, primarily on its S33 residue. This permits the proper and timely loading of RPA2 onto DNA and results in a safeguarding function by *PCAT19* that maintains the human endothelial cell resting state.

RESULTS

PCAT19 is highly enriched in endothelial cells and is differentially expressed in vascular diseases

When screening for endothelial lncRNAs in the FANTOM5 Cap Analysis of Gene Expression (CAGE) database,[2] we identified *PCAT19* as one of the most highly expressed lncRNAs in endothelial cells, with limited expression in other cell types (Figure 1A). Interestingly, *PCAT19* is listed in PanglaoDB as an endothelial marker.[32] Owing to its high endothelial expression, *PCAT19* is expressed in all human tissues listed in the GTEx database[1] (GTEx Analysis Release v.8; dbGaP accession phs000424.v8.p2). Tissues such as lung and spleen with a relatively dense vasculature, and therefore more endothelial cells, have the highest *PCAT19* expression compared with other tissues (Figure 1B). This tissue expression pattern was similar for other highly endothelial-enriched genes such as *CDH5* and *PECAM1* (Figure S1A). Given the initial identification of *PCAT19* in prostate tissue[33] and the elucidation of its role in prostate cancer,[34] we analyzed the expression of *PCAT19* in the prostate gland in more detail by interrogating publicly available data from a prostate single-cell RNA-sequencing (scRNA-seq) experiment (GEO: GSE172357).[5] In this unbiased dataset, *PCAT19* was highly enriched in the endothelial cell cluster with limited expression in other cell types (Figures 1C and 1D). The remarkable endothelial enrichment of *PCAT19* can also be observed in the Tabula Sapiens dataset.[9] The expression levels of *PCAT19*, *CDH5*, and *PECAM1* were compared across all human cell types. All three genes are clearly enriched in the endothelial cell cluster (Figure S1B). When looking at *PCAT19*, *CDH5*, and *PECAM1* expression in endothelial cells

(G) *PCAT19* expression relative to PECAM1 expression in stable (St) (n = 6) or unstable (Unst) (n = 5) carotid artery plaque samples from the Munich Vascular Biobank (30781475).
(H) Proportion of endothelial cells expressing *PCAT19* in lung scRNA-seq data.[7] Healthy endothelial cells (Norm), squamous cell carcinoma endothelial cells (SCC), and large-cell carcinoma endothelial cells (LCC) are shown. Data are presented as the mean ± SD; **p < 0.01, ***p < 0.001.

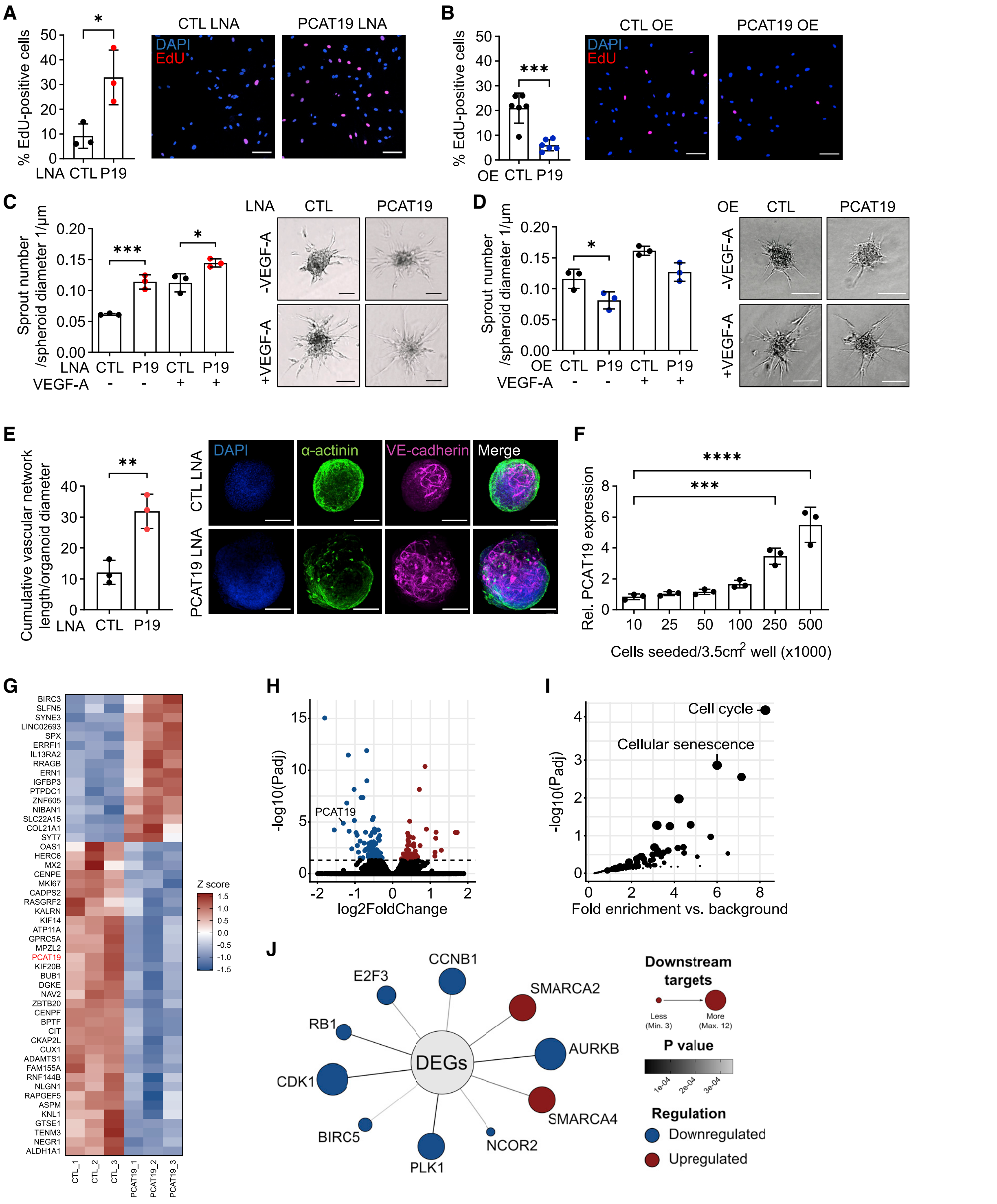

Figure 2. *PCAT19* represses endothelial cell proliferation, angiogenic sprouting, and cardiac organoid vascularization

(A) Endothelial cell proliferation measured by percentage EdU-positive cells after LNA GapmeR-mediated knockdown of *PCAT19* (P19) or negative control (CTL). Scale bars, 100 µm; n = 3 biological replicates, unpaired t test. Representative shown.

(B) Endothelial cell proliferation measured by percentage EdU-positive cells after overexpression (OE) of *PCAT19* (P19) or pcDNA3.1+ control (CTL). Scale bars, 100 µm; n = 6 biological replicates, unpaired t test. Representative images shown.

(legend continued on next page)

only and clustering by tissue, there is a clear widespread expression of each gene across endothelial cells from different tissues (Figure S1C).

Next, a possible impact of vascular diseases on *PCAT19* expression was sought by analyzing relevant RNA-seq datasets. Diseases of the vasculature often result in, or are caused by, differential rates of endothelial proliferation. *PCAT19* was significantly less expressed in hemangioma,[6] a malformation of blood vessels largely characterized by increased endothelial cell proliferation[35] (Figure 1E). In addition, in advanced carotid artery disease (characterized by plaque accumulation), *PCAT19* expression was significantly higher than in healthy or early disease samples (Figure 1F), but was similar between stable and unstable plaques from advanced carotid artery samples (Figure 1G). Due to the previous description of *PCAT19* in cancer, we checked whether the expression of *PCAT19* differed between healthy and cancerous tissues in the GEPIA database,[8] which returned a differential expression in most of the listed cancers, the majority of which displayed a downregulation of *PCAT19* in cancerous tissue compared with the respective healthy tissue (Figure S1D). This was most obvious in lung cancer samples (lung adenocarcinoma [LUAD] and lung squamous cell carcinoma [LUSC]) and highly intriguing. as lung tissue has the highest *PCAT19* expression in the GTEx data. Next, it was checked whether *PCAT19* expression differed specifically in the endothelial cells that formed a cancer compared with healthy endothelial cells. From publicly available lung scRNA-seq data[7] we observed that *PCAT19* was indeed expressed in fewer cancerous endothelial cells (squamous cell carcinoma [SCC] and large-cell carcinoma [LCC]) than in normal endothelial cells (Figure 1H). These data demonstrate not only a strong enrichment of *PCAT19* in endothelial cells but also its differential expression in vascular diseases and cancerous endothelial cells. This raises the question of what the functional significance of *PCAT19* is in endothelial cells.

PCAT19 represses proliferation, sprouting, and vascularization

Due to the enrichment of *PCAT19* in endothelial cells and its previously reported link to prostate cancer,[34] we wondered whether the perturbation of *PCAT19* would have an impact on endothelial cell cycle or growth. As determined by 5-ethynyl-2′-deoxyuridine (EdU) incorporation, the knockdown of *PCAT19* with LNA GapmeRs increased the rate of endothelial cell proliferation; 6 h after EdU application, three times as many cells had incorporated EdU after *PCAT19* knockdown compared with control cells (Figure 2A). Conversely, *PCAT19* overexpression by electroporation reduced endothelial cell proliferative capacity (Figure 2B). The *PCAT19* knockdown and overexpression efficiency are provided in Figure S1E. *PCAT19* knockdown using LNA GapmeRs was also performed in other endothelial cell types: human microvascular endothelial cells (HMECs), human carotid artery endothelial cells (HCAECs), human aortic endothelial cells (HAoECs), and human dermal lymphatic endothelial cells (HDLECs). Knockdown significantly promoted proliferation in HMECs and HAoECs but not in HCAECs or HDLECs, the latter of which did not proliferate well in general (Figure S2A). The effect of *PCAT19* perturbation on endothelial proliferation could also be confirmed using a CRISPRi and CRISPRa approach. *PCAT19* CRISPRi was able to promote endothelial proliferation, while *PCAT19* CRISPRa had the opposite effect in attenuating proliferation (Figure S2B). To further measure the relevance of *PCAT19* in endothelial growth and its potential impact on angiogenic sprouting, a three-dimensional endothelial spheroid outgrowth assay was performed. The knockdown of *PCAT19* promoted sprouting under both basal and VEGF-A-stimulated conditions (Figure 2C), while the overexpression of *PCAT19* attenuated sprouting under basal conditions (Figure 2D). Since *PCAT19* knockdown enhanced both endothelial proliferative and sprouting capacity, we hypothesized that a reduction in *PCAT19* levels may promote vascularization. This was studied in a three-dimensional organoid system, which involved the differentiation of induced pluripotent stem cells (iPSCs) into cardiomyocytes and endothelial cells to form functioning cardiac organoids. In this system, endothelial cells sprout and form contacts with neighboring endothelial sprouts, eventually forming a vascular network, with some vessels even containing a lumen.[36,37] All cardiac organoids formed a vascular network, but those subsequently transfected with *PCAT19* LNA GapmeRs produced a denser network, as measured by the cumulative vascular network length (Figure 2E). As *PCAT19* knockdown promoted cell cycle progression and proliferation, we wondered whether the expression of *PCAT19* itself is dependent on the cell proliferative state. Strikingly, *PCAT19* expression was strongly induced with cell density (as cells become more confluent and cell cycle arrested) (Figure 2F). These data demonstrate that *PCAT19* acts as an antiproliferative and antiangiogenic lncRNA that is induced during contact-mediated inhibition of the endothelial cell cycle.

To gain a deeper insight into how *PCAT19* may enact these antiproliferative and antiangiogenic effects, RNA-seq was performed after *PCAT19* knockdown. *PCAT19* itself was

(C and D) Endothelial cell spheroid outgrowth assay after LNA GapmeR-mediated knockdown of *PCAT19* (P19) or negative control (CTL) LNA GapmeR (C) or overexpression of *PCAT19* (P19) or pcDNA3.1+ control (CTL) (D). Spheroids were treated with and without VEGF-A. Scale bars, 100 μm; n = 3 biological replicates, one-way ANOVA. Representative images shown.

(E) Vascularization of cardiac organoids after LNA GapmeR-mediated knockdown of *PCAT19* (P19) or negative control (CTL). Scale bars, 200 μm; n = 3 biological replicates, unpaired t test. Representative images with maximum projection of the full z stack.

(F) *PCAT19* expression in HUVECs seeded at various densities; n = 3, one-way ANOVA.

(G) Heatmap of top 50 differentially expressed genes after *PCAT19* knockdown. The *Z* score is displayed (n = 3).

(H) Relative gene expression in response to *PCAT19* knockdown. Dashed line indicates a threshold of $p_{adj} < 0.05$ (n = 3).

(I) KEGG pathway fold enrichment over background from differentially expressed genes ($p_{adj} < 0.05$) after *PCAT19* knockdown.

(J) Prediction of upstream regulators of differentially expressed genes ($p_{adj} < 0.05$) after *PCAT19* knockdown using the QuaternaryProd R package. Color of outer circles indicates up- or downregulation for that upstream regulator. Size of circle indicates number of downstream targets. Thickness of line connecting inner and outer circles indicates significance level of that upstream regulator. Data are presented as the mean ± SD; *p < 0.05, **p < 0.01, ***p < 0.001, ****p < 0.0001.

significantly less expressed, confirming a successful knockdown (Figures 2G and 2H; Table S1). After filtering for differentially regulated genes ($p_{adj} < 0.05$), 186 genes were analyzed for Kyoto Encyclopedia of Genes and Genomes (KEGG) pathways. The top significant terms ($p_{adj} < 0.05$) were "cell cycle" and "cellular senescence" (Figure 2I), followed by "progesterone-mediated oocyte maturation" and "human T cell leukemia virus 1 infection" (Figures S2C and S2D). Some of the next terms, such as "MAPK signaling pathway," "transcriptional misregulation in cancer," and "p53 signaling pathway," are interesting and relevant, but were not significantly enriched with a $p_{adj} < 0.05$. We therefore decided to focus on the top term, "cell cycle." The same group of 186 differentially regulated genes was used to identify their potential upstream regulators using the QuaternaryProd package. The top 10 predicted regulators were mapped according to their number of significant downstream targets and whether the regulators themselves were up- or downregulated (Figures 2J and S2E). Most of these, such as CCNB1, E2F3, PLK1, and CDK1, are strongly involved in cell cycle and senescence,[38] confirming that *PCAT19* indeed has a profound impact on cell cycle. Since *PCAT19* seems to be important for endothelial proliferation and angiogenic sprouting, coupled with the associated expression changes in cell cycle genes upon *PCAT19* knockdown, we chose to further investigate the role of *PCAT19* in cell cycle regulation, given the indication from the RNA-seq experiment that "cell cycle" is affected to some degree by *PCAT19* knockdown.

PCAT19 binds the DNA replication protein A complex

The biological effects of lncRNAs are often mediated through their interaction with other RNAs, DNA, or proteins. Since *PCAT19* had a profound effect on the cell cycle, we wondered whether this resulted from a potential interaction of *PCAT19* with cell-cycle-related proteins. We first determined the subcellular localization of *PCAT19* using RNA fluorescence *in situ* hybridization (FISH) and noticed a large fraction of *PCAT19* localized to the nucleus (Figure S3A), which would at least place it within close proximity to cell cycle proteins. Cytoplasmic and nuclear fractionation of endothelial cells revealed an equal distribution of *PCAT19* between the cytoplasm and the nucleus under subconfluent conditions (Figure S3B). Surprisingly, there was significantly more *PCAT19* localized to the nucleus compared with the cytoplasm under confluent conditions, again highlighting the potential importance of *PCAT19* in the nucleus. To determine whether *PCAT19* indeed interacts with proteins, the endogenously expressed *PCAT19* was pulled down using biotinylated antisense oligonucleotides (AS-oligos) containing a *PCAT19*-specific targeting sequence. Mass spectrometry identified eight significantly enriched ($p < 0.05$) proteins in the *PCAT19* pull-down versus scramble control pull-down (Figures 3A and 3B; Table S2). Using the $\log_{10}$ intensity-based absolute quantitation (iBAQ) value, the most abundant of the eight *PCAT19*-enriched proteins were RPA1, RPA2, and RPA3: the three members of the RPA complex. DNA ligase 3 (LIG3) and its known interaction partner X-ray repair cross complementing 1 (XRCC1) were also enriched with *PCAT19*. In addition, ubiquitin-like with PHD and ring finger domain 1 (UHRF1) and UHRF2 as well as polynucleotide kinase 3′-phosphatase (PNKP) were identified as *PCAT19* interaction partners. Each of these proteins is involved in the DNA damage response, DNA replication, or the cell cycle.[17,39–41] Given that these proteins interact with DNA and, in some cases, with one another (Figure 3C), the primary *PCAT19* interactor could not be inferred from this experiment alone. The proteins of the RPA complex (RPA1, 2, and 3) were the most abundant of the enriched interactors. Of these, RPA2 can be considered the central target of regulatory pathways, as it is subject to extensive regulation through dynamic and sequential context-dependent phosphorylation on several sites.[18] A potential interaction between RPA2 and *PCAT19* was therefore investigated in more detail. The *PCAT19*-RPA2 interaction was confirmed with AS-oligo pull-down from endothelial cell lysates and Western blotting (Figure 3D). The interaction was further confirmed with an immunoprecipitation of RPA2 followed by RNA isolation and qRT-PCR (RIP-qPCR) for *PCAT19* (Figure 3E). The 18S rRNA and U4 snRNA were not enriched with RPA2, as expected. To exclude that these findings were a consequence of an indirect interaction through other proteins tightly bound to RPA2, a fully *in vitro* approach was used with purified His-tagged RPA2 incubated with or without *in vitro*-transcribed biotinylated *PCAT19*. Pull-down of biotinylated *PCAT19* recovered His-tagged RPA2, demonstrating that the interaction between the two molecules is indeed direct (Figure 3F). The central role of RPA2 in DNA repair and synthesis processes and the role of *PCAT19* in limiting cellular proliferation may suggest that *PCAT19* mediates its effects through RPA2 in a cell-cycle- or DNA damage-dependent manner.

Loss of PCAT19 predisposes and sensitizes DNA to damage

Given that the RPA complex and the other *PCAT19*-interacting proteins are involved in the DNA damage and repair response, the potential contribution of *PCAT19* to this process was determined. Interestingly, after *PCAT19* knockdown, cells displayed a positive terminal deoxynucleotidyl transferase dUTP nick-end labeling (TUNEL) signal indicative of DNA double-strand breaks, which could not be detected in the cells transfected with control LNA GapmeRs (Figure 4A). Upon treatment with camptothecin (CPT), an inducer of DNA double-strand breaks, cells displayed a noticeable increase in TUNEL signal, and this was exacerbated by LNA directed against *PCAT19*. This suggests that the loss of *PCAT19* may lead to an accumulation of DNA damage. A comet assay confirmed these findings, as *PCAT19* knockdown resulted in a significantly longer tail olive moment compared with cells transfected with control LNA (Figure 4B). In line with this, knockdown of *PCAT19* enhanced the DNA damage-induced accumulation of p53 and γH2AX (Figures 4C and 4D), whereas *PCAT19* overexpression had the opposite effect (Figures 4E and 4F). We also observed the LNA GapmeR-mediated knockdown of *PCAT19* to increase p53 levels in HCAECs, HAoECs, HMECs, and HDLECs (Figure S3C). Again, this was more pronounced after treatment with CPT. The same was true for *PCAT19* knockdown on S33-pRPA2 levels in these other endothelial cell types (Figure S3D). The effects on p53 levels in the presence and absence of *PCAT19* could also be confirmed with the CRISPRi and CRISPRa approach. *PCAT19* CRISPRi was able to increase p53 levels after CPT stimulation (Figure 4G), while CRISPRa reduced p53 levels

CellPress
OPEN ACCESS

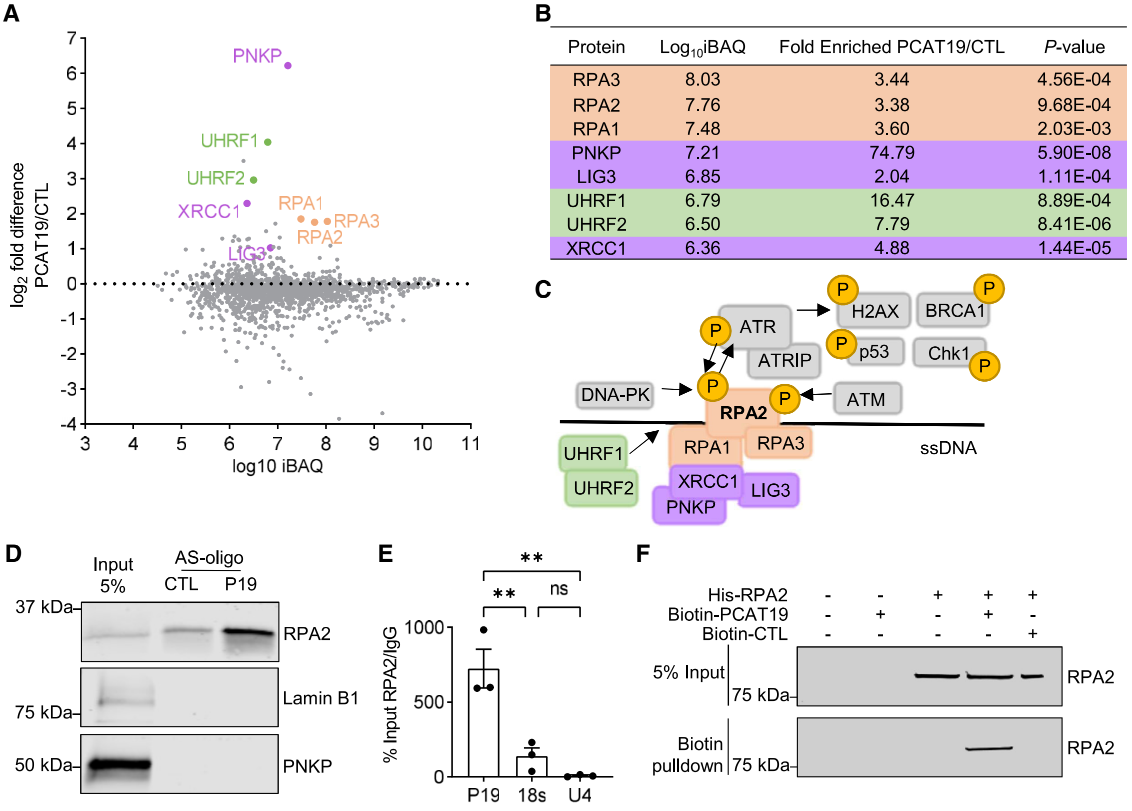

Protein	Log_{10}iBAQ	Fold Enriched PCAT19/CTL	*P*-value
RPA3	8.03	3.44	4.56E-04
RPA2	7.76	3.38	9.68E-04
RPA1	7.48	3.60	2.03E-03
PNKP	7.21	74.79	5.90E-08
LIG3	6.85	2.04	1.11E-04
UHRF1	6.79	16.47	8.89E-04
UHRF2	6.50	7.79	8.41E-06
XRCC1	6.36	4.88	1.44E-05

Figure 3. RPA2 is a *PCAT19* interaction partner

(A) Biotin-tagged antisense-oligo (AS-oligo) RNA pull-down of *PCAT19* and its interacting proteins from endothelial cell lysate measured by mass spectrometry. Scrambled AS-oligos were used as negative control (CTL). A log_{10}iBAQ representation of enriched proteins (sum of all peptide intensities/number of observable peptides) against the log_2 fold difference of *PCAT19*/CTL is shown (n = 6). Highlighted proteins indicate enrichment with *PCAT19* ($p < 0.05$; $q < 0.05$). iBAQ, intensity-based absolute quantitation.

(B) Table of significantly enriched *PCAT19*-interacting proteins ($p < 0.05$, $q < 0.05$).

(C) Schematic depicting the proteins pulled down with *PCAT19* and potential interaction map based on literature searches. RPA2 is central to the RPA complex and reportedly functions alongside most of the proteins identified with mass spectrometry.

(D) AS-oligo RNA pull-down of *PCAT19* (P19) or control AS-oligos (CTL) and western blot with antibodies against RPA2 and PKNP. Lamin B1 served as negative control.

(E) RNA immunoprecipitation (RIP) in HUVEC extract with an antibody against RPA2 followed by qRT-PCR for *PCAT19*. Percentage of input recovery of *PCAT19* versus a non-primary-antibody control (IgG) is shown. 18S rRNA and U4 snRNA served as negative controls.

(F) *In vitro* binding assay of RPA2 and *PCAT19*. His-tagged RPA2 was combined with *in vitro*-transcribed and biotinylated *PCAT19* or pcDNA3.1+ control RNA (biotin-CTL). Streptavidin beads were used to pull down the biotin-tagged RNAs and blots stained for RPA2. Data are presented as the mean ± SD; **$p < 0.01$.

after CPT stimulation (Figure 4H). Next, an RNA *in situ* hybridization followed by a proximity ligation assay (rISH-PLA) was performed to determine whether *PCAT19* co-localized with γH2AX and where in the cell this co-localization occurs. The biotin-tagged antisense-oligonucleotides specific to *PCAT19*, an antibody against biotin, and an antibody against yH2AX were used. A conventional PLA was performed with secondary antibodies against the primary biotin and yH2AX antibodies. *PCAT19* and yH2AX indeed co-localized in nuclear foci as visualized by positive PLA signals, and the numbers of interaction sites significantly increased after treatment with CPT (Figures 4I and S3E). RPA is central in DNA synthesis and homologous recombination where it binds ssDNA and prevents the formation of secondary DNA structures that could impede DNA replication or repair.[17] To determine whether *PCAT19*, via its interaction with RPA2, had an impact on DNA replication, a DNA fiber assay was performed. Despite reducing the rate of cell proliferation, *PCAT19* overexpression had no effect on DNA replication speed, as indicated by similar DNA tract lengths between *PCAT19* and pcDNA3.1+ control-overexpressing cells (Figure S2F).

Given the increased rate of endothelial cell proliferation and accumulation of DNA damage after *PCAT19* knockdown, we wondered whether cell cycle transitions themselves were affected by *PCAT19* knockdown. A bromodeoxyuridine (BrdU) incorporation and propidium iodide staining followed by fluorescence-activated cell sorting (FACS) analysis was performed after *PCAT19* knockdown, but these cells did not display a difference in cell cycle phase profiles (Figure 4J). However, treatment with hydroxyurea (HU), which causes replication stress, led to a significantly greater accumulation of cells in early S phase (S1) and significantly fewer cells in the G2/M phase after *PCAT19* knockdown compared with control cells (Figure 4J). Given that

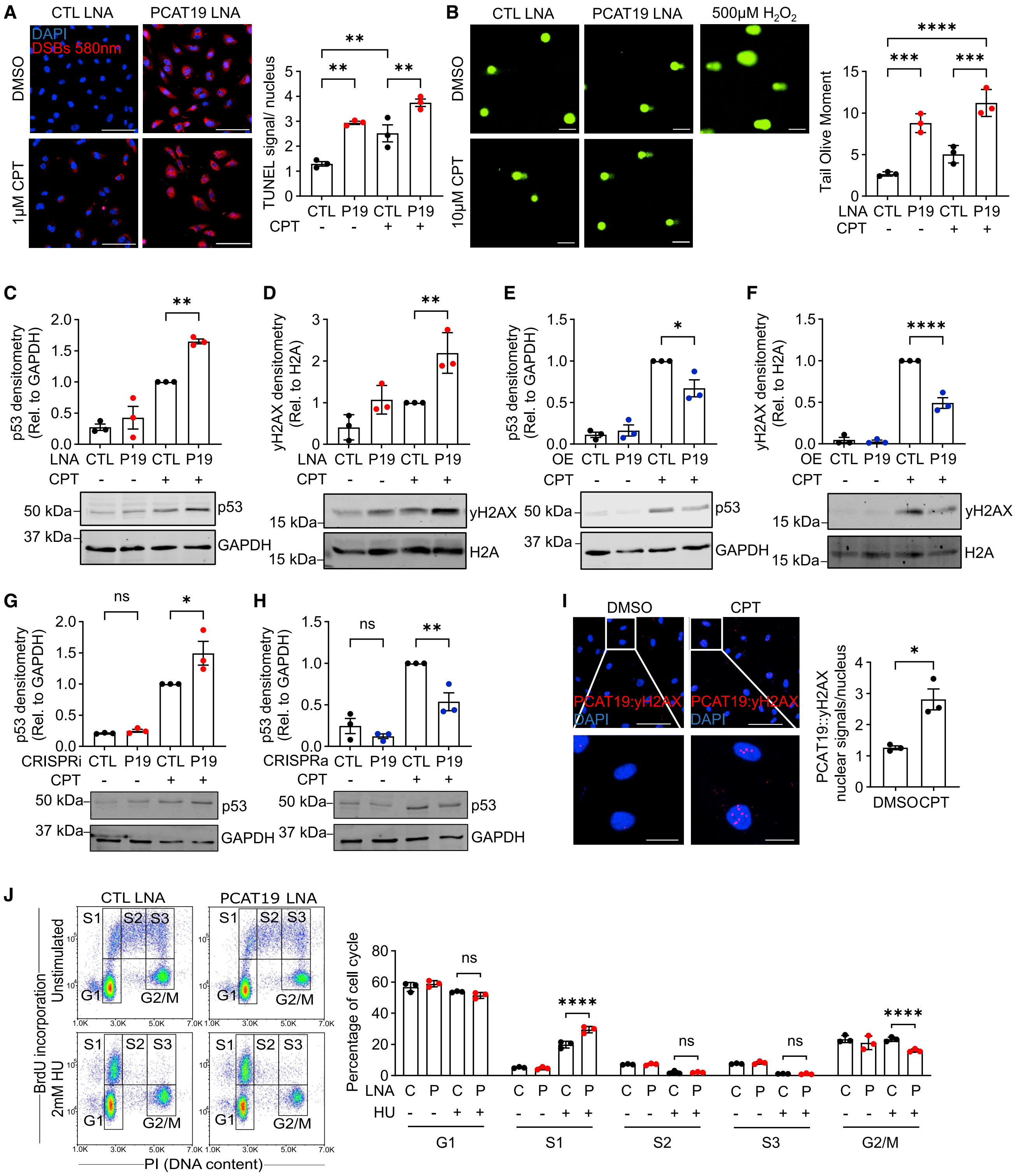

Figure 4. *PCAT19* maintains genomic stability and limits RPA2-ATR signaling

(A) HUVECs were transfected with LNA GapmeRs against *PCAT19* or negative control LNA and treated with 1 μM camptothecin or DMSO for 16 h. TUNEL assay was performed and cells were imaged for DNA double-strand breaks. Double-strand breaks are shown in red (Alexa Fluor 580 nm). DAPI was used to stain nuclei (blue). Scale bars, 100 μm. Quantification of TUNEL signal mean intensity per nucleus is shown. One-way ANOVA; error bars show the mean ± SD; n = 3 biological replicates.

(legend continued on next page)

there was no difference between control and *PCAT19* knockdown cells in middle and late S phase, the increased accumulation of *PCAT19* knockdown cells in early S phase presumably arises from the G2/M population. This highlights the faster transitioning through cell cycle after *PCAT19* knockdown, and in this case from G2/M back to G1, and thereby a sensitization of *PCAT19* knockdown cells to DNA damage.

PCAT19 protects RPA2 from uncontrolled phosphorylation

The phosphorylation of RPA2 on its S33 residue is a tightly controlled process mediated by the ATR kinase that precedes cell cycle transition from S phase into G2 phase[18] (Figure 5A). S33-pRPA2 is required for the efficient repair of ssDNA that may have been produced from damaged DNA during replication.[42] As RPA2 phosphorylation in endothelial cells has not been studied, S33-pRPA2 levels were compared between proliferating subconfluent and non-proliferating confluent human umbilical vein endothelial cells (HUVECs). Proliferating cells exhibited higher S33-pRPA2 levels than growth-arrested cells and, as expected, ATRi massively reduced S33-pRPA2 levels under both conditions (Figure 5B). Surprisingly, *PCAT19*-knockdown cells exhibited significantly elevated S33-pRPA2 levels (Figure 5C), while the overexpression of *PCAT19* reduced S33-pRPA2 levels (Figure 5D). In all knockdown and overexpression conditions, additional ATR inhibition markedly reduced S33-pRPA2 levels, as expected (Figures 5B–5D). To determine whether S33 phosphorylation affects the interaction between RPA2 and *PCAT19*, a semi-*in vitro* binding assay was performed with recombinant His-RPA2 and *in vitro*-transcribed *PCAT19* in the presence of ssDNA. HUVEC lysate was added to the mixture to permit RPA2 phosphorylation by kinases. RPA2 and *PCAT19* were again found to strongly interact and, unexpectedly, this interaction could be blocked by ATR inhibitor or phosphatase treatment (Figure 5E). Since the phosphorylation of S4/8-RPA2 occurs after the phosphorylation of S33-RPA2, we tested whether S4/8-pRPA2 levels would be altered in the presence of *PCAT19*. Another semi-*in vitro* assay was performed, this time in HEK293 lysate, where FLAG-ATR and His-RPA2 were added with or without *in vitro*-transcribed *PCAT19*. With the addition of FLAG-ATR to the lysate containing His-RPA2, more S4/8-pRPA2 was formed (since ssDNA was also present in the mixture to promote RPA2 loading and phosphorylation). Importantly, both the His-tagged RPA2 and the endogenous RPA2 were phosphorylated on S4/8. As expected, *PCAT19* was able to strongly attenuate both the endogenous and the His-tagged RPA2 S4/8 phosphorylation levels (Figure 5F). These results suggest that *PCAT19* can bind to and modulate S33-pRPA2 and prevent the sequential hyperphosphorylation of RPA2, as measured by lower S4/8-pRPA2 levels. This particular assay was also performed in HEK293T lysate, where *PCAT19* should not be present, to confirm the molecular action of *PCAT19* on RPA2.

Since *PCAT19* seems to have an effect primarily on ATR-dependent RPA2 S33 phosphorylation, we wondered whether *PCAT19* mediates the interaction between ATR and RPA2. Indeed, *PCAT19* overexpression attenuated the interaction between RPA2 and ATR, as determined by PLA (Figure 5G). In addition, the ATRi was able to reverse *PCAT19* knockdown-induced proliferation (Figure 5H) and angiogenic sprouting (Figure 5I), indicating that this growth phenotype after *PCAT19* knockdown is related to elevated p-RPA2.

Taken together, these results demonstrate a regulatory role for *PCAT19* in endothelial S33-RPA2 phosphorylation, which ultimately controls the state of downstream sequential RPA2 hyperphosphorylation. *PCAT19* reduces the degree of RPA2-ATR interaction and the levels of phosphorylation of the ATR target, S33-RPA2. S33-pRPA2 is required for proliferation; thus, the hyperproliferation resulting from *PCAT19* knockdown is a consequence of increased ATR-dependent S33-pRPA2 phosphorylation. Importantly, premature hyperphosphorylation of RPA2 that is not bound to DNA prevents its subsequent loading onto DNA and thereby an inability to efficiently repair DNA damage.[22,24] Therefore, depletion of *PCAT19* promotes the uncontrolled hyperphosphorylation of RPA2, rendering it unable to repair DNA damage or signal for cell cycle arrest; this leads to the observed phenotype of endothelial hyperproliferation and DNA damage accumulation.

DISCUSSION

We have identified *PCAT19* as a highly enriched endothelial lncRNA that is induced by quiescence to fine-tune and protect

(B) HUVECs were transfected with LNA GapmeRs against *PCAT19* or negative control LNA and treated with 10 μM camptothecin or DMSO for 16 h. Comet assay was performed and cells were imaged. Quantification of comets and tail olive moment is shown (n = 3). Scale bars, 100 μm.

(C and D) HUVECs were transfected with *PCAT19* (P19) LNA or negative control (CTL) LNA and then treated with or without CPT. Western blot staining for (C) p53 and GAPDH or (D) yH2AX and H2A (n = 3 for all).

(E and F) HUVECs were transduced with either *PCAT19* (P19) overexpression (OE) plasmid or pcDNA3.1+ backbone control (CTL) plasmid and then treated with or without camptothecin (CPT). Western blot staining for (E) p53 and GAPDH or (F) yH2AX and H2A (n = 3 for all).

(G) HUVECs were transfected with *PCAT19* CRISPRi or respective negative controls and treated with or without CPT for 16 h. Western blot staining for p53 and GAPDH; n = 3 biological replicates.

(H) HUVECs were transfected with *PCAT19* CRISPRa or respective negative controls and treated with or without CPT for 16 h. Western blot staining for p53 and GAPDH; n = 3 biological replicates.

(I) RNA *in situ* hybridization proximity ligation assay (rISH-PLA) between *PCAT19* and γH2AX treated with DMSO or CPT. Biotin-tagged *PCAT19* antisense oligonucleotides and antibodies against biotin and γH2AX were added to fixed cells. Cells that received only *PCAT19* oligonucleotides/biotin antibody or yH2AX antibody served as negative controls. Red signal indicates PLA signal (546 nm) between *PCAT19* and γH2AX, blue indicates DAPI. Scale bars, top, 100 μm; bottom, 25 μm.

(J) HUVECs were transfected with LNA GapmeRs against *PCAT19* (P) or negative control LNA (C) and treated with and without 2 mM HU for 16 h. Cells were analyzed by FACS after BrdU incorporation and propidium iodide staining. Cell cycle phases are indicated. Quantification for percentage cells in each phase (G1, S1, S2, S3, and G2/M) is displayed (n = 3). Data are presented as the mean ± SD; *$p < 0.05$, **$p < 0.01$, ***$p < 0.001$, ****$p < 0.0001$.

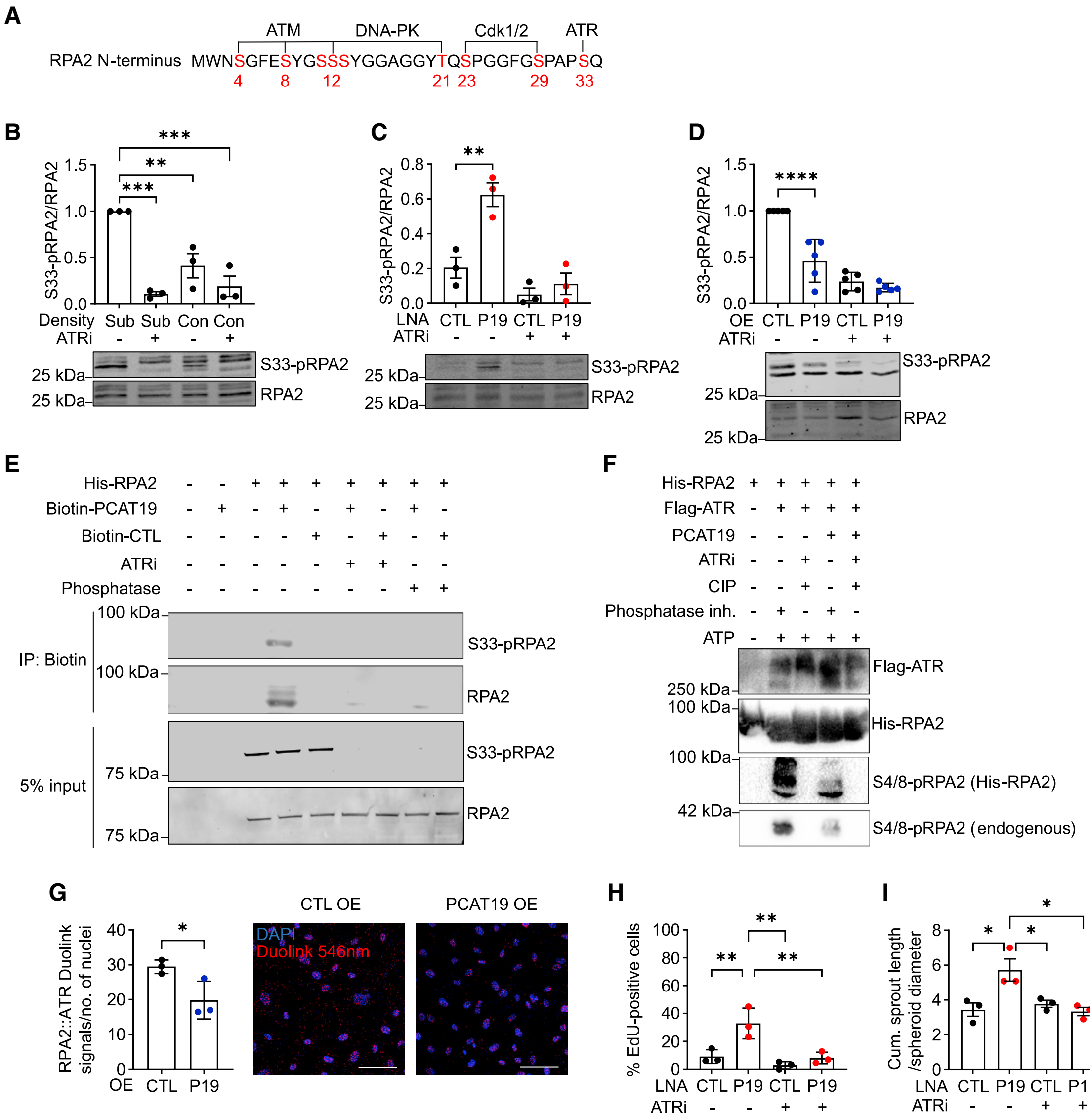

Figure 5. *PCAT19* limits RPA2 serine 33 (S33) phosphorylation

(A) Depiction of RPA2 phosphorylation sites.

(B) HUVECs were seeded at subconfluent or confluent levels and treated with 10 μM ATRi or DMSO for 16 h. Western blot staining for RPA2 and S33-pRPA2 is shown (n = 3).

(C) HUVECs were transfected with LNA GapmeRs against *PCAT19* or negative control LNA and treated with 10 μM ATRi or DMSO for 16 h. Western blot staining for RPA2 and S33-pRPA2 is shown (n = 3).

(D) HUVECs were transduced with either *PCAT19* overexpression (OE) plasmid or pcDNA3.1+ backbone control plasmid and then treated with 10 μM ATRi or DMSO for 16 h. Western blot staining for RPA2 and S33-pRPA2 is shown (n = 6).

(E) *In vitro* binding assay for various combinations of His-RPA2, biotin-*PCAT19*, biotin-CTL RNA, ATRi, and phosphatase. Staining of S33-pRPA2 or RPA2 in biotin pull-down and 5% input samples is shown.

(F) *In vitro* phosphorylation assay of endogenous RPA2 and recombinant His-RPA2. Combinations of His-RPA2, FLAG-ATR, *in vitro*-transcribed *PCAT19*, ATR inhibitor, phosphatase (CIP), phosphatase inhibitor, and ATP are shown. FLAG, His, and S4/8-pRPA2 antibodies were used for staining.

(G) HUVECs were transduced with either *PCAT19* overexpression (OE) plasmid or pcDNA3.1+ backbone control plasmid. Duolink proximity ligation assay for RPA2-ATR is shown. Red signal indicates the Duolink PLA signal (546 nm), blue indicates DAPI.

(H) EdU proliferation assay after *PCAT19* LNA-GapmeR-mediated knockdown or control LNA and with or without treatment with 10 μM ATRi for 16 h (n = 3).

(I) Spheroid outgrowth assay after *PCAT19* LNA-GapmeR-mediated knockdown or control LNA and with or without treatment with 10 μM ATRi for 16 h (n = 3).

Data are presented as the mean ± SD; *p < 0.05, **p < 0.01, ***p < 0.001, ****p < 0.0001.

CellPress
OPEN ACCESS

RPA2 from excessive phosphorylation. In doing so, *PCAT19* aids in the slowing of the cell cycle and inhibition of angiogenic sprouting while safeguarding the DNA of endothelial cells during the proliferation-quiescence switch. When *PCAT19* is knocked down, RPA2 can be prematurely and excessively phosphorylated, which influences its cell cycle and DNA damage condition-dependent functionality. This ultimately results in cell cycle promotion and hyperproliferation with an overall reduced DNA stability.

More than 100 lncRNAs were originally identified as being strongly associated with prostate cancer and subsequently termed the prostate cancer-associated transcripts (PCATs).[33,43] A handful of studies have characterized some of the PCATs in more detail, one of which reported the importance of *PCAT19* in the development of cancer,[34,44] but did not address its physiological function in health. We were surprised to find that such a prominent cancer-related lncRNA was so highly enriched in healthy endothelial cells. The strong induction of *PCAT19* with endothelial contact inhibition of the cell cycle, taken together with its previously described roles in cancer, suggested that *PCAT19* could maintain certain aspects of endothelial quiescence. This quiescent state is particularly important for long-lived endothelial cells to maintain the functioning inner monolayer of blood vessels.[45] Our data indicate that *PCAT19* facilitates DNA integrity and repair, which is required for long-lived, non-dividing cells. On the other hand, endothelial cells require the ability to rapidly re-enter the cell cycle and proliferate under conditions that either damage the blood vessels or promote angiogenesis. This behavior of the endothelium is a somewhat unique cellular feature. For example, in epithelial cells, healing is facilitated by increased proliferation of progenitor cells, while mesenchymal cellular activation results in an expansion of an undifferentiated cell pool (like fibroblasts) rather than a transient activation. Thus, *PCAT19*, which is differentially expressed between single and confluent cells, may therefore have specifically evolved to address the conflicting needs of rapidly proliferating and long-lived endothelial phenotypes. This not only explains its endothelial-specific expression but also may help to explain why *PCAT19* is a human-specific lncRNA. Humans have a relatively long lifespan compared with other mammals and therefore have a need to balance cell proliferation and repair and maintenance of DNA integrity. Indeed, genomic instability is one of the main causative factors of vascular aging, which itself is a risk factor for cardiovascular disease.

While the study of Hua et al.[34] highlighted an SNP risk region within the *PCAT19* locus that ultimately mediates prostate cancer progression, we have identified a specific role for *PCAT19* in the quiescent-proliferative switch of human endothelial cells. Hua et al. demonstrated a reduced proliferation of cancer cells after *PCAT19* knockdown, while we observed an increased proliferation of endothelial cells accompanied by the accumulation of spontaneous DNA damage. It is becoming abundantly clear that lncRNAs have evolved cell-type-specific functions and mechanisms of action; this includes certain lncRNAs that are highly and ubiquitously expressed. The lncRNA *H19*, for example, interacts with HuR in epithelial cells to regulate barrier function,[46] with methyl-CpG-binding domain protein 1 (MBD1) in mouse embryonic fibroblasts to mediate embryonic growth,[47] and with p53 to inhibit apoptosis in gastric cancer cells.[48] The exact regulatory mechanisms between lncRNAs and their protein interaction partners in different cell types are not completely understood. This is likely due to a complex interplay between cell-type-specific transcription factors, the expression of lncRNAs themselves, and their downstream molecular targets. We uncovered the RPA complex as the strongest *PCAT19* interactor in endothelial cells, again pointing toward a fundamental role in cell cycle regulation, specifically in DNA stability and cell cycle checkpoints. This goes hand-in-hand with our RNA-seq of HUVECs that returned "cell cycle" as the top term after *PCAT19* knockdown; this supports the finding that *PCAT19* is upregulated with cell cycle arrest, and its removal promotes cell cycle re-entry.

Once bound to ssDNA, RPA acts as a platform to recruit and regulate multiple other protein factors essential for DNA stability and maintenance. Since the genome is constantly exposed to different types of DNA damage, the coordination of cell cycle and DNA damage-response proteins is of paramount importance for an appropriate and measured response. Faulty checkpoint activation can result in uncontrolled growth and irreparable DNA damage, which often triggers cell apoptosis. However, if the damage occurs within oncogenes, tumor-suppressor genes, or genes that control the cell cycle, then cancer can develop.[49] Knockdown of *PCAT19* promoted proliferation and angiogenic sprouting, and this was accompanied by a heightened sensitivity to DNA-damaging agents such as CPT and HU. Owing to the strong binding of *PCAT19* to the RPA complex, we hypothesized that the role of *PCAT19* in endothelial quiescence and apparent safeguarding of the genome could be mediated directly through its interaction with RPA2.

RPA2 is phosphorylated on the S33 residue at the beginning of S phase and is then dephosphorylated upon the successful completion of mitosis. RPA2 can also undergo sequential hyperphosphorylation by three PI3K-like protein kinases (ATR, ATM, and DNA-PK) depending on the type and level of DNA damage. For example, the resection of DNA double-strand breaks promotes the phosphorylation of RPA2 by ATR on the S33 residue; this then permits the subsequent phosphorylation at S4/8 by DNA-PKs. If functional ATR is missing, ssDNA accumulates from DNA resection and leads to the exhaustion of RPA pools. As such, ATR phosphorylation of RPA2 S33 aids in the prevention of ssDNA accumulation.[18] If hyperphosphorylation of DNA-bound RPA occurs, a signaling cascade is activated that ultimately leads to cell cycle arrest and activation of the DNA damage response. However, it is important to note that if premature hyperphosphorylation of RPA2 occurs, the RPA complex does not bind as efficiently to DNA and therefore damage can accumulate.[22,24] This offers a potential explanation for the increased levels of DNA damage following *PCAT19* knockdown: depletion of *PCAT19* promotes the uncontrolled hyperphosphorylation of RPA2, which could prevent its efficient binding to DNA in the repair response and a potential lack of cell cycle arrest signals. This may then lead to cell cycle progression and the accumulation of DNA damage.

It has also already been shown that the phosphorylation of RPA2 is a dynamic process rather than a simple "on-off" phosphorylative switch. Lee and colleagues showed that RPA2 must

undergo phosphorylation followed by rapid de-phosphorylation by human protein phosphatase 4 (PP4) for the successful repair of double-strand breaks.[50] Depletion of PPR4 leads to an extended G2-M checkpoint and the accumulation of DNA damage. This lends support to the hypothesis that *PCAT19* could also function as a mediator that fine-tunes RPA2 phosphorylation. *PCAT19* knockdown heightened the levels of DNA damage and promoted S33 phosphorylation by increasing the interaction between RPA2 and ATR, the kinase responsible for S33 phosphorylation. These results suggest that *PCAT19* binds RPA2 and protects it from excessive S33 phosphorylation by ATR in a cell-cycle-dependent manner. We confirmed that subconfluent proliferating endothelial cells have heightened levels of S33-pRPA2, as would be expected in cycling cells. However, when *PCAT19* is knocked down, S33-pRPA2 levels increase further to maintain genome stability during a faster cell cycle progression as seen with the hyperproliferative response. This excessive S33 phosphorylation could equally disable RPA2 and negatively affect DNA damage responses. Interestingly, our *in vitro* binding experiments revealed that addition of an ATR inhibitor or a phosphatase could abolish the *PCAT19*-RPA2 interaction. This suggests a model in which *PCAT19* may bind RPA2 to fine-tune the levels of S33 phosphorylation in the presence of phosphorylated and active ATR. S33 phosphorylation is a precursor to S4/8 phosphorylation, the hallmark of RPA2 phosphorylation, which should therefore be dependent on *PCAT19*-S33-pRPA2 modulation. Indeed, S4/8-pRPA2 levels were markedly reduced in the presence of *PCAT19*. Importantly, this semi-*in vitro* assay for S4/8-pRPA2 was performed using HEK293 lysate, indicating that the molecular mechanism would be ubiquitous. However, the endothelial enrichment of *PCAT19* ensures that this particular regulation of RPA2 is restricted to endothelial cells. Of course, inhibition or removal of ATR prevents S33 phosphorylation and so *PCAT19* may be removed from RPA2 to permit S33 phosphorylation and avoid faulty DNA damage-repair responses. Importantly, ATR inhibition was able to rescue the *PCAT19* knockdown-induced increase in proliferation and angiogenic sprouting, supporting the idea that the heightened S33-pRPA2 levels permit cell cycling.

Under conditions that damage the vessel or promote new vessel growth, endothelial cells re-enter the cell cycle to reach confluence again. This quiescent-proliferative switch is central in many vascular diseases. For example, infantile hemangioma, which is the most common type of tumor in infants, results from increased proliferation of endothelial cells and pericytes.[35] It was therefore interesting to find a significant and marked reduction in *PCAT19* expression in hemangioma samples. The opposite endothelial proliferative scenario is often observed in carotid artery restenosis and atherosclerosis,[51,52] characterized by damage to the endothelium, reduced proliferation, and formation of a neointima, which is essentially scar tissue on the inner blood vessel. In this scenario, *PCAT19* is significantly more highly expressed; this again correlates with the endothelial proliferative rate. At first glance, the concept that endothelial cells favor rapid proliferation over tight control of DNA integrity is surprising. It is, however, important to mention that endothelial proliferation due to faulty contact inhibition is a somewhat rare event in mature vessels and occurs only at sites of vessel damage.

PCAT19 could play a role in many vascular diseases that depend on the proliferation of endothelial cells, as well as in tumor angiogenesis, which is crucial in supporting cancer growth. It is therefore tempting to speculate that, by targeting *PCAT19* and thereby influencing the fine-tuning of the S33-pRPA2 switch, the endothelial quiescence-proliferation transition could be controlled to positively alter the outcome of vascular disease. In conclusion, with the present work, we identified the endothelial-enriched lncRNA *PCAT19*, which safeguards the endothelial genome by interacting with and modulating RPA2. Upon loss of contact inhibition, for example, during vascular injury, *PCAT19* expression decreases and thereby facilitates rapid endothelial monolayer repair by permitting normal RPA2 phosphorylation.

Limitations of the study

The current study highlights the cell-type- and condition-specific functions of lncRNAs by providing new insights into the fine-tuning of RPA2 phosphorylation in endothelial cells by the lncRNA *PCAT19*. Our data demonstrate that *PCAT19* binds RPA2, alters the RPA2 phosphorylation state, and influences cell cycle progression and DNA damage responses. Details on the precise *PCAT19*-RPA2 interaction are missing; the exact binding site of *PCAT19* on RPA2 and whether binding at this site physically prevents kinase accessibility are unclear and would require mutagenesis experiments and structural analyses. The dynamics of this interaction and in particular the promotion of binding factors and the subsequent inhibition of binding are unknown. We propose that the endothelial enrichment of *PCAT19* is what confers this endothelial-specific mechanism of RPA2 regulation. It is unclear as to whether this mechanism exists between RPA2 and lncRNAs in other cell types. Although we provide data on the differential expression of *PCAT19* in vascular diseases and in cancer endothelial cells, exactly how *PCAT19* is involved in these diseases is so far unknown. Evidence in human vascular disease cohorts is sparse, and the use of an *in vivo* model is not possible due to the lack of *PCAT19* conservation between species. The transcriptional regulation of *PCAT19* needs to be clarified, since it has been reported in cancer cells, and we report its enrichment in healthy endothelial cells.

STAR★METHODS

Detailed methods are provided in the online version of this paper and include the following:

- KEY RESOURCES TABLE
- RESOURCE AVAILABILITY
  - Lead contact
  - Materials availability
  - Data and code availability
- EXPERIMENTAL MODEL AND SUBJECT DETAILS
  - Primary cell cultures and cell lines
- METHOD DETAILS
  - Cell stimulations
  - LNA GapmeR-mediated knockdown
  - Overexpression
  - EdU proliferation assay

CellPress
OPEN ACCESS

  - ○ Spheroid outgrowth assay
  - ○ Human cardiac organoid formation
  - ○ DNA fiber assay
  - ○ Overexpression and purification of RPA2 proteins
  - ○ *In vitro* transcription and RNA 3′end biotinylation
  - ○ PCAT19-RPA2 *in vitro* assays
  - ○ RNA isolation, reverse transcription and RT-qPCR
  - ○ Protein isolation and western blot by SDS-PAGE
  - ○ RNA immunoprecipitation
  - ○ Antisense-oligonucleotide pulldown of RNA
  - ○ CRISPR/dCas9 activation (CRISPRa) and inactivation (CRISPRi)
  - ○ Mass spectrometry
  - ○ Proximity ligation assay (PLA)
  - ○ RNA-fluorescent *in situ* hybridisation (FISH)
  - ○ RNA *in situ* hybridization-proximity ligation assay (rISH-PLA)
  - ○ Terminal deoxynucleotidyl transferase dUTP nick end labeling (TUNEL)
  - ○ Comet assay
  - ○ BrdU/PI FACS
  - ○ RNA-sequencing
  - ○ Human carotid artery plaques
- QUANTIFICATION AND STATISTICAL ANALYSIS

SUPPLEMENTAL INFORMATION

Supplemental information can be found online at https://doi.org/10.1016/j.celrep.2022.111670.

ACKNOWLEDGMENTS

We thank Jana Meisterknecht for help with mass spectrometry, Thomas Böttger for help with the rISH-PLA method, Eva Wolf for help with RPA purification, and Nicolas Jaé for sharing RNA protein pull-down protocols. Support from the IMB Microscopy Core Facility is gratefully acknowledged. This work was supported by the Goethe University Frankfurt am Main, the DFG Excellence Cluster Cardiopulmonary Institute (CPI) EXS2026, the Dr. Rolf M. Schwiete Stiftung, and the DFG Transregio TRR267 (Project-ID 403584255-TRR 267; TP A04, TP A06, TP B04, TP B07, and TP Z02). A.H.B. is supported by the British Heart Foundation Chair of Translational Cardiovascular Sciences. Work in I.D. lab is supported by the grants from Else Kroener Fresenius Stiftung and Dr. Rolf M. Schwiete Stiftung. R.P.B. and A.H.B. are supported by the BHF/DHF/DZHK grant "Exploiting endothelial long non-coding RNAs to promote regenerative angiogenesis in the damaged myocardium."

AUTHOR CONTRIBUTIONS

Conceptualization, J.A.O., M.S.L., and R.P.B.; methodology, J.A.O., T.W., I.W., C.P.G., V.M., I.T., K. Petriukov, L.M., Z.W., J.K., M.D.P., S.G., M.S.L., and R.P.B.; formal analysis, J.A.O., T.W., I.W., L.M., Z.W., C.P.G., S.G., and M.S.L.; investigation, J.A.O., K. Pálfi, T.W., I.W., C.P.G., V.M., I.T., F.B., L.M., Z.W., J.K., M.D.P., J.I.P., T.T., K. Petriukov, S.H., S.G., and M.S.L.; data curation, J.A.O., T.W., I.W., S.G., and M.S.L.; visualization, J.A.O., T.W., C.P.G., and M.S.L.; resources, L.M., and Z.W.; writing – original draft, J.A.O., M.S.L., and R.P.B.; writing – review & editing, J.A.O., M.S.L., and R.P.B.; supervision, A.H.B., H.D.U., I.D., M.S.L., and R.P.B.; funding acquisition, A.H.B., M.S.L., and R.P.B.

DECLARATION OF INTERESTS

The authors declare no competing interests.

Received: March 29, 2022
Revised: August 18, 2022
Accepted: September 24, 2022
Published: November 15, 2022

REFERENCES

1. GTEx Consortium (2020). The GTEx Consortium atlas of genetic regulatory effects across human tissues. Science *369*, 1318–1330. https://doi.org/10.1126/science.aaz1776.
2. Lizio, M., Harshbarger, J., Shimoji, H., Severin, J., Kasukawa, T., Sahin, S., Abugessaisa, I., Fukuda, S., Hori, F., Ishikawa-Kato, S., et al. (2015). Gateways to the FANTOM5 promoter level mammalian expression atlas. Genome Biol. *16*, 22. https://doi.org/10.1186/s13059-014-0560-6.
3. Noguchi, S., Arakawa, T., Fukuda, S., Furuno, M., Hasegawa, A., Hori, F., Ishikawa-Kato, S., Kaida, K., Kaiho, A., Kanamori-Katayama, M., et al. (2017). FANTOM5 CAGE profiles of human and mouse samples. Sci. Data *4*, 170112. https://doi.org/10.1038/sdata.2017.112.
4. FANTOM Consortium and the RIKEN PMI and CLST DGT; Forrest, A.R.R., Kawaji, H., Rehli, M., Baillie, J.K., de Hoon, M.J.L., Haberle, V., Lassmann, T., Kulakovskiy, I.V., Lizio, M., et al. (2014). A promoter-level mammalian expression atlas. Nature *507*, 462–470. https://doi.org/10.1038/nature13182.
5. Joseph, D.B., Henry, G.H., Malewska, A., Reese, J.C., Mauck, R.J., Gahan, J.C., Hutchinson, R.C., Malladi, V.S., Roehrborn, C.G., Vezina, C.M., and Strand, D.W. (2021). Single-cell analysis of mouse and human prostate reveals novel fibroblasts with specialized distribution and microenvironment interactions. J. Pathol. *255*, 141–154. https://doi.org/10.1002/path.5751.
6. Li, X., Chen, Y., Fu, C., Li, H., Yang, K., Bi, J., and Huo, R. (2020). Characterization of epigenetic and transcriptional landscape in infantile hemangiomas with ATAC-seq and RNA-seq. Epigenomics *12*, 893–905. https://doi.org/10.2217/epi-2020-0060.
7. Goveia, J., Rohlenova, K., Taverna, F., Treps, L., Conradi, L.-C., Pircher, A., Geldhof, V., de Rooij, L.P.M.H., Kalucka, J., Sokol, L., et al. (2020). An integrated gene expression landscape profiling approach to identify lung tumor endothelial cell heterogeneity and angiogenic candidates. Cancer Cell *37*, 21–36.e13. https://doi.org/10.1016/j.ccell.2019.12.001.
8. Tang, Z., Li, C., Kang, B., Gao, G., Li, C., and Zhang, Z. (2017). GEPIA: a web server for cancer and normal gene expression profiling and interactive analyses. Nucleic Acids Res. *45*, W98–W102. https://doi.org/10.1093/nar/gkx247.
9. Tabula Sapiens Consortium; Jones, R.C., Karkanias, J., Krasnow, M.A., Pisco, A.O., Quake, S.R., Salzman, J., Yosef, N., Bulthaup, B., Brown, P., et al. (2022). The Tabula Sapiens: a multiple-organ, single-cell transcriptomic atlas of humans. Science *376*, eabl4896. https://doi.org/10.1126/science.abl4896.
10. Potente, M., and Mäkinen, T. (2017). Vascular heterogeneity and specialization in development and disease. Nat. Rev. Mol. Cell Biol. *18*, 477–494. https://doi.org/10.1038/nrm.2017.36.
11. Li, X., Sun, X., and Carmeliet, P. (2019). Hallmarks of endothelial cell metabolism in health and disease. Cell Metab. *30*, 414–433. https://doi.org/10.1016/j.cmet.2019.08.011.
12. Ricard, N., Bailly, S., Guignabert, C., and Simons, M. (2021). The quiescent endothelium: signalling pathways regulating organ-specific endothelial normalcy. Nat. Rev. Cardiol. *18*, 565–580. https://doi.org/10.1038/s41569-021-00517-4.
13. Grazia Lampugnani, M., Zanetti, A., Corada, M., Takahashi, T., Balconi, G., Breviario, F., Orsenigo, F., Cattelino, A., Kemler, R., Daniel, T.O., and Dejana, E. (2003). Contact inhibition of VEGF-induced proliferation requires vascular endothelial cadherin, beta-catenin, and the phosphatase DEP-1/CD148. J. Cell Biol. *161*, 793–804. https://doi.org/10.1083/jcb.200209019.

14. Suzuki, E., Nagata, D., Yoshizumi, M., Kakoki, M., Goto, A., Omata, M., and Hirata, Y. (2000). Reentry into the cell cycle of contact-inhibited vascular endothelial cells by a phosphatase inhibitor. Possible involvement of extracellular signal-regulated kinase and phosphatidylinositol 3-kinase. J. Biol. Chem. *275*, 3637–3644. https://doi.org/10.1074/jbc.275.5.3637.

15. Giannotta, M., Trani, M., and Dejana, E. (2013). VE-cadherin and endothelial adherens junctions: active guardians of vascular integrity. Dev. Cell *26*, 441–454. https://doi.org/10.1016/j.devcel.2013.08.020.

16. Panagopoulos, A., and Altmeyer, M. (2021). The hammer and the dance of cell cycle control. Trends Biochem. Sci. *46*, 301–314. https://doi.org/10.1016/j.tibs.2020.11.002.

17. Zou, Y., Liu, Y., Wu, X., and Shell, S.M. (2006). Functions of human replication protein A (RPA): from DNA replication to DNA damage and stress responses. J. Cell. Physiol. *208*, 267–273. https://doi.org/10.1002/jcp.20622.

18. Vassin, V.M., Anantha, R.W., Sokolova, E., Kanner, S., and Borowiec, J.A. (2009). Human RPA phosphorylation by ATR stimulates DNA synthesis and prevents ssDNA accumulation during DNA-replication stress. J. Cell Sci. *122*, 4070–4080. https://doi.org/10.1242/jcs.053702.

19. Maréchal, A., and Zou, L. (2015). RPA-coated single-stranded DNA as a platform for post-translational modifications in the DNA damage response. Cell Res. *25*, 9–23. https://doi.org/10.1038/cr.2014.147.

20. Olson, E., Nievera, C.J., Klimovich, V., Fanning, E., and Wu, X. (2006). RPA2 is a direct downstream target for ATR to regulate the S-phase checkpoint. J. Biol. Chem. *281*, 39517–39533. https://doi.org/10.1074/jbc.M605121200.

21. Fanning, E., Klimovich, V., and Nager, A.R. (2006). A dynamic model for replication protein A (RPA) function in DNA processing pathways. Nucleic Acids Res. *34*, 4126–4137. https://doi.org/10.1093/nar/gkl550.

22. Oakley, G.G., Patrick, S.M., Yao, J., Carty, M.P., Turchi, J.J., and Dixon, K. (2003). RPA phosphorylation in mitosis alters DNA binding and protein-protein interactions. Biochemistry *42*, 3255–3264. https://doi.org/10.1021/bi026377u.

23. Fang, F., and Newport, J.W. (1993). Distinct roles of cdk2 and cdc2 in RP-A phosphorylation during the cell cycle. J. Cell Sci. *106* (*Pt 3*), 983–994. https://doi.org/10.1242/jcs.106.3.983.

24. Lai, Y., Zhu, M., Wu, W., Rokutanda, N., Togashi, Y., Liang, W., and Ohta, T. (2019). HERC2 regulates RPA2 by mediating ATR-induced Ser33 phosphorylation and ubiquitin-dependent degradation. Sci. Rep. *9*, 14257. https://doi.org/10.1038/s41598-019-50812-x.

25. Jain, A.K. (2020). Emerging roles of long non-coding RNAs in the p53 network. RNA Biol. *17*, 1648–1656. https://doi.org/10.1080/15476286.2020.1770981.

26. Ghafouri-Fard, S., Shoorei, H., Anamag, F.T., and Taheri, M. (2020). The role of non-coding RNAs in controlling cell cycle related proteins in cancer cells. Front. Oncol. *10*, 608975. https://doi.org/10.3389/fonc.2020.608975.

27. Khanduja, J.S., Calvo, I.A., Joh, R.I., Hill, I.T., and Motamedi, M. (2016). Nuclear noncoding RNAs and genome stability. Mol. Cell *63*, 7–20. https://doi.org/10.1016/j.molcel.2016.06.011.

28. Oo, J.A., Brandes, R.P., and Leisegang, M.S. (2022). Long non-coding RNAs: novel regulators of cellular physiology and function. Pflugers Arch. *474*, 191–204. https://doi.org/10.1007/s00424-021-02641-z.

29. Statello, L., Guo, C.-J., Chen, L.-L., and Huarte, M. (2021). Gene regulation by long non-coding RNAs and its biological functions. Nat. Rev. Mol. Cell Biol. *22*, 96–118. https://doi.org/10.1038/s41580-020-00315-9.

30. Wang, L., Li, J., Zhou, H., Zhang, W., Gao, J., and Zheng, P. (2021). A novel lncRNA Discn fine-tunes replication protein A (RPA) availability to promote genomic stability. Nat. Commun. *12*, 5572. https://doi.org/10.1038/s41467-021-25827-6.

31. Flynn, R.L., Centore, R.C., O'Sullivan, R.J., Rai, R., Tse, A., Songyang, Z., Chang, S., Karlseder, J., and Zou, L. (2011). TERRA and hnRNPA1 orchestrate an RPA-to-POT1 switch on telomeric single-stranded DNA. Nature *471*, 532–536. https://doi.org/10.1038/nature09772.

32. Franzén, O., Gan, L.-M., and Björkegren, J.L.M. (2019). PanglaoDB: a web server for exploration of mouse and human single-cell RNA sequencing data. Database *2019*, baz046. https://doi.org/10.1093/database/baz046.

33. Prensner, J.R., Iyer, M.K., Balbin, O.A., Dhanasekaran, S.M., Cao, Q., Brenner, J.C., Laxman, B., Asangani, I.A., Grasso, C.S., Kominsky, H.D., et al. (2011). Transcriptome sequencing across a prostate cancer cohort identifies PCAT-1, an unannotated lincRNA implicated in disease progression. Nat. Biotechnol. *29*, 742–749. https://doi.org/10.1038/nbt.1914.

34. Hua, J.T., Ahmed, M., Guo, H., Zhang, Y., Chen, S., Soares, F., Lu, J., Zhou, S., Wang, M., Li, H., et al. (2018). Risk SNP-mediated promoter-enhancer switching drives prostate cancer through lncRNA PCAT19. Cell *174*, 564–575.e18. https://doi.org/10.1016/j.cell.2018.06.014.

35. Ji, Y., Chen, S., Li, K., Li, L., Xu, C., and Xiang, B. (2014). Signaling pathways in the development of infantile hemangioma. J. Hematol. Oncol. *7*, 13. https://doi.org/10.1186/1756-8722-7-13.

36. Wagner, J.U.G., Pham, M.D., Nicin, L., Hammer, M., Bottermann, K., Yuan, T., Sharma, R., John, D., Muhly-Reinholz, M., Tombor, L., et al. (2020). Dissection of heterocellular cross-talk in vascularized cardiac tissue mimetics. J. Mol. Cell. Cardiol. *138*, 269–282. https://doi.org/10.1016/j.yjmcc.2019.12.005.

37. Liu, C., Oikonomopoulos, A., Sayed, N., and Wu, J.C. (2018). Modeling human diseases with induced pluripotent stem cells: from 2D to 3D and beyond. Development *145*, dev156166. https://doi.org/10.1242/dev.156166.

38. Giacinti, C., and Giordano, A. (2006). RB and cell cycle progression. Oncogene *25*, 5220–5227. https://doi.org/10.1038/sj.onc.1209615.

39. Gao, Y., Katyal, S., Lee, Y., Zhao, J., Rehg, J.E., Russell, H.R., and McKinnon, P.J. (2011). DNA ligase III is critical for mtDNA integrity but not Xrcc1-mediated nuclear DNA repair. Nature *471*, 240–244. https://doi.org/10.1038/nature09773.

40. Luo, T., Cui, S., Bian, C., and Yu, X. (2013). Uhrf2 is important for DNA damage response in vascular smooth muscle cells. Biochem. Biophys. Res. Commun. *441*, 65–70. https://doi.org/10.1016/j.bbrc.2013.10.018.

41. Dumitrache, L.C., and McKinnon, P.J. (2017). Polynucleotide kinase-phosphatase (PNKP) mutations and neurologic disease. Mech. Ageing Dev. *161*, 121–129. https://doi.org/10.1016/j.mad.2016.04.009.

42. Shi, W., Feng, Z., Zhang, J., Gonzalez-Suarez, I., Vanderwaal, R.P., Wu, X., Powell, S.N., Roti Roti, J.L., Gonzalo, S., and Zhang, J. (2010). The role of RPA2 phosphorylation in homologous recombination in response to replication arrest. Carcinogenesis *31*, 994–1002. https://doi.org/10.1093/carcin/bgq035.

43. Ylipää, A., Kivinummi, K., Kohvakka, A., Annala, M., Latonen, L., Scaravilli, M., Kartasalo, K., Leppänen, S.P., Karakurt, S., Seppälä, J., et al. (2015). Transcriptome sequencing reveals PCAT5 as a novel ERG-regulated long noncoding RNA in prostate cancer. Cancer Res. *75*, 4026–4031. https://doi.org/10.1158/0008-5472.CAN-15-0217.

44. Zhang, X., Wang, Q., Xu, Y., Wang, B., Jia, C., Wang, L., Sun, H., Zhao, H., Wang, Z., Zou, Q., et al. (2019). lncRNA PCAT19 negatively regulates p53 in non-small cell lung cancer. Oncol. Lett. *18*, 6795–6800. https://doi.org/10.3892/ol.2019.11041.

45. Suzuki, E., Nishimatsu, H., Nagata, D., Satonaka, H., Goto, A., Omata, M., Fujita, T., Nagai, R., and Hirata, Y. (2002). Constitutive activation of proto-oncogen protein p21 induces cell cycle arrest in the G1 phase in contact-inhibited vascular endothelial cells. Hypertens. Res. *25*, 773–778. https://doi.org/10.1291/hypres.25.773.

46. Zou, T., Jaladanki, S.K., Liu, L., Xiao, L., Chung, H.K., Wang, J.-Y., Xu, Y., Gorospe, M., and Wang, J.-Y. (2016). H19 long noncoding RNA regulates intestinal epithelial barrier function via MicroRNA 675 by interacting with RNA-binding protein HuR. Mol. Cell Biol. *36*, 1332–1341. https://doi.org/10.1128/MCB.01030-15.

47. Monnier, P., Martinet, C., Pontis, J., Stancheva, I., Ait-Si-Ali, S., and Dandolo, L. (2013). H19 lncRNA controls gene expression of the Imprinted Gene Network by recruiting MBD1. Proc. Natl. Acad. Sci. USA *110*, 20693–20698. https://doi.org/10.1073/pnas.1310201110.

48. Yang, F., Bi, J., Xue, X., Zheng, L., Zhi, K., Hua, J., and Fang, G. (2012). Up-regulated long non-coding RNA H19 contributes to proliferation of gastric cancer cells. FEBS J. *279*, 3159–3165. https://doi.org/10.1111/j.1742-4658.2012.08694.x.

49. Gabrielli, B., Brooks, K., and Pavey, S. (2012). Defective cell cycle checkpoints as targets for anti-cancer therapies. Front. Pharmacol. *3*, 9. https://doi.org/10.3389/fphar.2012.00009.

50. Lee, D.-H., Pan, Y., Kanner, S., Sung, P., Borowiec, J.A., and Chowdhury, D. (2010). A PP4 phosphatase complex dephosphorylates RPA2 to facilitate DNA repair via homologous recombination. Nat. Struct. Mol. Biol. *17*, 365–372. https://doi.org/10.1038/nsmb.1769.

51. Xu, S., Ilyas, I., Little, P.J., Li, H., Kamato, D., Zheng, X., Luo, S., Li, Z., Liu, P., Han, J., et al. (2021). Endothelial dysfunction in atherosclerotic cardiovascular diseases and beyond: from mechanism to pharmacotherapies. Pharmacol. Rev. *73*, 924–967. https://doi.org/10.1124/pharmrev.120.000096.

52. Kipshidze, N., Dangas, G., Tsapenko, M., Moses, J., Leon, M.B., Kutryk, M., and Serruys, P. (2004). Role of the endothelium in modulating neointimal formation: vasculoprotective approaches to attenuate restenosis after percutaneous coronary interventions. J. Am. Coll. Cardiol. *44*, 733–739. https://doi.org/10.1016/j.jacc.2004.04.048.

53. Cortez, D., Guntuku, S., Qin, J., and Elledge, S.J. (2001). ATR and ATRIP: partners in checkpoint signaling. Science *294*, 1713–1716. https://doi.org/10.1126/science.1065521.

54. Kearns, N.A., Genga, R.M.J., Enuameh, M.S., Garber, M., Wolfe, S.A., and Maehr, R. (2014). Cas9 effector-mediated regulation of transcription and differentiation in human pluripotent stem cells. Development *141*, 219–223. https://doi.org/10.1242/dev.103341.

55. Konermann, S., Brigham, M.D., Trevino, A.E., Joung, J., Abudayyeh, O.O., Barcena, C., Hsu, P.D., Habib, N., Gootenberg, J.S., Nishimasu, H., et al. (2015). Genome-scale transcriptional activation by an engineered CRISPR-Cas9 complex. Nature *517*, 583–588. https://doi.org/10.1038/nature14136.

56. Schindelin, J., Arganda-Carreras, I., Frise, E., Kaynig, V., Longair, M., Pietzsch, T., Preibisch, S., Rueden, C., Saalfeld, S., Schmid, B., et al. (2012). Fiji: an open-source platform for biological-image analysis. Nat. Methods *9*, 676–682. https://doi.org/10.1038/nmeth.2019.

57. Doench, J.G., Fusi, N., Sullender, M., Hegde, M., Vaimberg, E.W., Donovan, K.F., Smith, I., Tothova, Z., Wilen, C., Orchard, R., et al. (2016). Optimized sgRNA design to maximize activity and minimize off-target effects of CRISPR-Cas9. Nat. Biotechnol. *34*, 184–191. https://doi.org/10.1038/nbt.3437.

58. Tyanova, S., Temu, T., and Cox, J. (2016). The MaxQuant computational platform for mass spectrometry-based shotgun proteomics. Nat. Protoc. *11*, 2301–2319. https://doi.org/10.1038/nprot.2016.136.

59. Tyanova, S., Temu, T., Sinitcyn, P., Carlson, A., Hein, M.Y., Geiger, T., Mann, M., and Cox, J. (2016). The Perseus computational platform for comprehensive analysis of (prote)omics data. Nat. Methods *13*, 731–740. https://doi.org/10.1038/nmeth.3901.

60. Andrews, S. (2010). FastQC: A Quality Control Tool for High Throughput Sequence Data.

61. Bolger, A.M., Lohse, M., and Usadel, B. (2014). Trimmomatic: a flexible trimmer for Illumina sequence data. Bioinformatics *30*, 2114–2120. https://doi.org/10.1093/bioinformatics/btu170.

62. Dobin, A., Davis, C.A., Schlesinger, F., Drenkow, J., Zaleski, C., Jha, S., Batut, P., Chaisson, M., and Gingeras, T.R. (2013). STAR: ultrafast universal RNA-seq aligner. Bioinformatics *29*, 15–21. https://doi.org/10.1093/bioinformatics/bts635.

63. Liao, Y., Smyth, G.K., and Shi, W. (2014). featureCounts: an efficient general purpose program for assigning sequence reads to genomic features. Bioinformatics *30*, 923–930. https://doi.org/10.1093/bioinformatics/btt656.

64. Love, M.I., Huber, W., and Anders, S. (2014). Moderated estimation of fold change and dispersion for RNA-seq data with DESeq2. Genome Biol. *15*, 550. https://doi.org/10.1186/s13059-014-0550-8.

65. Fakhry, C.T., Choudhary, P., Gutteridge, A., Sidders, B., Chen, P., Ziemek, D., and Zarringhalam, K. (2016). Interpreting transcriptional changes using causal graphs: new methods and their practical utility on public networks. BMC Bioinf. *17*, 318. https://doi.org/10.1186/s12859-016-1181-8.

66. Wu, T., Hu, E., Xu, S., Chen, M., Guo, P., Dai, Z., Feng, T., Zhou, L., Tang, W., Zhan, L., et al. (2021). clusterProfiler 4.0: a universal enrichment tool for interpreting omics data. Innovation *2*, 100141. https://doi.org/10.1016/j.xinn.2021.100141.

67. Yu, G., and He, Q.-Y. (2016). ReactomePA: an R/Bioconductor package for reactome pathway analysis and visualization. Mol. Biosyst. *12*, 477–479. https://doi.org/10.1039/c5mb00663e.

68. Wickham, H. (2016). ggplot2. Elegant Graphics for Data Analysis (Springer International Publishing).

69. Korff, T., and Augustin, H.G. (1998). Integration of endothelial cells in multicellular spheroids prevents apoptosis and induces differentiation. J. Cell Biol. *143*, 1341–1352. https://doi.org/10.1083/jcb.143.5.1341.

70. Rappsilber, J., Mann, M., and Ishihama, Y. (2007). Protocol for micro-purification, enrichment, pre-fractionation and storage of peptides for proteomics using StageTips. Nat. Protoc. *2*, 1896–1906. https://doi.org/10.1038/nprot.2007.261.

71. Schwanhäusser, B., Busse, D., Li, N., Dittmar, G., Schuchhardt, J., Wolf, J., Chen, W., and Selbach, M. (2011). Global quantification of mammalian gene expression control. Nature *473*, 337–342. https://doi.org/10.1038/nature10098.

72. Roussis, I.M., Myers, F.A., and Scarlett, G.P. (2017). RNA whole-mount in situ hybridization proximity ligation assay (rISH-PLA), an assay for detecting RNA-protein complexes in intact cells. Curr. Protoc. Cell Biol. *74*, 17.20.1–1-17.20.10. https://doi.org/10.1002/cpcb.13.

73. Leisegang, M.S., Bibli, S.-I., Günther, S., Pflüger-Müller, B., Oo, J.A., Höper, C., Seredinski, S., Yekelchyk, M., Schmitz-Rixen, T., Schürmann, C., et al. (2019). Pleiotropic effects of laminar flow and statins depend on the Krüppel-like factor-induced lncRNA MANTIS. Eur. Heart J. *40*, 2523–2533. https://doi.org/10.1093/eurheartj/ehz393.

74. Fasolo, F., Jin, H., Winski, G., Chernogubova, E., Pauli, J., Winter, H., Li, D.Y., Glukha, N., Bauer, S., Metschl, S., et al. (2021). Long noncoding RNA MIAT controls advanced atherosclerotic lesion formation and plaque destabilization. Circulation *144*, 1567–1583. https://doi.org/10.1161/CIRCULATIONAHA.120.052023.

75. Redgrave, J.N.E., Lovett, J.K., Gallagher, P.J., and Rothwell, P.M. (2006). Histological assessment of 526 symptomatic carotid plaques in relation to the nature and timing of ischemic symptoms: the Oxford plaque study. Circulation *113*, 2320–2328. https://doi.org/10.1161/CIRCULATIONAHA.105.589044.

76. Kontos, C., El Bounkari, O., Krammer, C., Sitinski, D., Hille, K., Zan, C., Yan, G., Wang, S., Gao, Y., Brandhofer, M., et al. (2020). Designed CXCR4 mimic acts as a soluble chemokine receptor that blocks atherogenic inflammation by agonist-specific targeting. Nat. Commun. *11*, 5981. https://doi.org/10.1038/s41467-020-19764-z.

STAR★METHODS

KEY RESOURCES TABLE

REAGENT or RESOURCE	SOURCE	IDENTIFIER
Antibodies		
anti-BrdU, mouse	BD Biosciences	347580; RRID:AB_400326
anti-BrdU, rat	Abcam	ab6326; RRID:AB_305426
Anti-RPA32/RPA2 antibody [9H8], mouse	Abcam	ab2175; RRID:AB_302873
Rabbit anti-RPA32 Antibody	Bethyl	A300-244A; RRID:AB_185548
Anti-phospho-RPA32 (Ser33), rabbit	Bethyl	A300-246A; RRID:AB_2180847
Anti-RPA32/RPA2 (phospho T21) antibody, rabbit	Abcam	ab61065; RRID:AB_946322
Anti-phospho-RPA32 (Ser4/Ser8) Recombinant Monoclonal, rabbit	Bethyl	A700-009; RRID:AB_2765278
ATR antibody (C-1)	Santa Cruz	sc-515173; RRID:AB_2893291
p53 antibody (FL-393), rabbit	Santa Cruz	sc-6243; RRID:AB_653753
Lamin B1 antibody (H-90), rabbit	Santa Cruz	sc-20682; RRID:AB_2136308
PNK1 Polyclonal Antibody (PNKP), rabbit	Bethyl	A300-257A; RRID:AB_263356
Anti-phospho-Histone H2A.X (Ser139) Antibody, rabbit	Millipore	MABE205; RRID:AB_10851746
Histone H2A (L88A6) Mouse mAb	Cell Signaling Technology	3636; RRID:AB_2118801
Monoclonal Anti-α-Actinin (Sarcomeric) antibody produced in mouse	Sigma-Aldrich	A7811; RRID:AB_476766
VE-Cadherin (D87F2) XP® Rabbit mAb	Cell Signaling Technology	2500; RRID:AB_10839118
DYKDDDDK Tag (D6W5B) Rabbit mAb (Binds to same epitope as Sigma's Anti-FLAG® M2 Antibody)	Cell Signaling Technology	14793; RRID:AB_2572291
Anti-His6	Roche	11922416001; RRID:AB_514486
Anti-GAPDH antibody, Mouse monoclonal	Sigma-Aldrich	G8795; RRID:AB_1078991
Anti-β-Actin antibody, Mouse monoclonal	Sigma-Aldrich	A1978; RRID:AB_476692
Anti-Biotin antibody [Hyb-8]	Abcam	ab201341; RRID:AB_2861249
Bacterial and virus strains		
NEB Turbo Competent E. coli (High Efficiency)	NEB	C2984H
Biological samples		
Pooled human umbilical vein endothelial cells (HUVEC)	PromoCell	C-12203; Lot No.: 474Z010, 408Z014, 471Z011, 466Z022
human coronary artery endothelial cells (HCAEC)	PeloBiotech	PB-CH-182-2011; Lot No. QC06814F10
human aortic endothelial cells (HAoEC)	PeloBiotech	304K-05a; Lot No. 2366
Human dermal lymphatic endothelial cells (HDLEC)	Promocell	C-12217; Lot No. 394Z027.3, 4092401.3
Human cardiac organoids (hCOs)	This study	N/A
Chemicals, peptides, and recombinant proteins		
Recombinant Human VEGF 165 Protein	R&D	293-VE; Accession # NP_001165097
(S)-(+)-Camptothecin	Sigma-Aldrich	C9911; CAS: 7689-03-4
VE-821 ATR inhibitor	Selleckchem	S8007; CAS: 1232410-49-9
Hydroxyurea	Sigma-Aldrich	H-8627; CAS: 127-07-1
Phosphatase, Alkaline, Calf Intestine	Merck	524572; CAS: 9001-78-9
5-Bromo-2-deoxyuridine (BrdU)	Roche	10280879001; CAS: 59-14-3

(Continued on next page)

Continued

REAGENT or RESOURCE	SOURCE	IDENTIFIER
Critical commercial assays		
STEMdiff™ Cardiomyocyte Differentiation Kit	STEMCELL Technologies	05010
Pierce RNA 3'end biotinylation kit	ThermoFisher	20160
CometAssay Single Cell Gel Electrophoresis Assay	R&D Systems	4250-050-K
SMARTer Stranded Total RNA Sample Prep Kit - HI Mammalian	Takara	634873
Deposited data		
RNA-Seq PCAT19 knockdown data	This paper	GEO: GSE199091
Raw mass spectrometric data of PCAT19 protein interaction partners	This paper	PRIDE: PXD032669
Experimental models: Cell lines		
Human microvascular endothelial cells (HMEC)	CDC	98247
293T/17 [HEK 293T/17] (HEK293T)	ATCC	CRL-11268; RRID:CVCL_1926
293 [HEK-293]	ATCC	CRL-1573; RRID:CVCL_0045
Human induced pluripotent stem cells (hIPSCs)	EbiSC	WSTIi081-A
Oligonucleotides		
LNA GapmeR *PCAT19* 5′-AATTCGGCTCTTACAA-3′	This study	N/A
Primers for 18S rRNA, GAPDH, PCAT19 and U4 snRNA, see Table S1	This study	N/A
PCAT19 antisense-oligonucleotide (5′-Biotin-AAG CAG ACA TGA GAC CTC ACT-3′)	This study	N/A
scramble control oligonucleotide (5′-Biotin-GTG TAA CAC GTC TAT ACG CCC A-3′)	This study	N/A
PCAT19 antisense-oligonucleotide (5′-TYE665-AAG CAG ACA TGA GAC CTC ACT-3′)	This study	N/A
scramble control oligonucleotide (5′-TYE665-GTG TAA CAC GTC TAT ACG CCC A-3′)	This study	N/A
Recombinant DNA		
Plasmid: pcDNA3.1 + *PCAT19*	This study	N/A
Plasmid: pcDNA3.1+	ThermoFisher	V79020
Plasmid: CMV Flag ATRwt	Cortez et al., 2001[53]	Addgene plasmid #41909
Plasmid: pHAGE EF1α dCas9-VP64	Kearns et al., 2014[54]	Addgene plasmid #50918
Plasmid: pHAGE EF1α dCas9-KRAB	Kearns et al., 2014[54]	Addgene plasmid #50919
Plasmid: sgRNA(MS2) vector	Konermann et al., 2015[55]	Addgene plasmid #61424
Plasmid: sgRNA(MS2) vector-CRISPRa-PCAT19_gRNA	This study	N/A
Plasmid: sgRNA(MS2) vector-CRISPRi-PCAT19_gRNA	This study	N/A
Software and algorithms		
FIJI/ImageJ	Schindelin et al., 2012[56]	RRID:SCR_002285
Leica LAS X	Leica Microsystems	RRID:SCR_013673
Image Studio Ver 5.2	Licor	RRID:SCR_015795

(*Continued on next page*)

Continued

REAGENT or RESOURCE	SOURCE	IDENTIFIER
CRISPick GPP sgRNA designer	Doench et al., 2016[57]	https://portals.broadinstitute.org/gppx/crispick/public
MaxQuant 1.6.1.0132	Tyanova et al., 2016[58]	RRID:SCR_014485
Perseus 1.6.1.3	Tyanova et al., 2016[59]	RRID:SCR_015753
CometScore 2.0	TriTek Corp	http://rexhoover.com/index.php?id=cometscore
FlowJo v10.8	BD Life Sciences	RRID:SCR_008520
FastQC	Andrews, 2010[60]	RRID:SCR_014583
Trimmomatic 0.39	Bolger et al., 2014[61]	RRID:SCR_011848
STAR 2.74.9a	Dobin et al., 2013[62]	RRID:SCR_004463
featureCounts 2.0.2	Liao et al., 2014[63]	RRID:SCR_012919
DESeq2 1.30.1	Love et al., 2014[64]	RRID:SCR_015687
QuaternaryProd	Fakhry et al., 2016[65]	https://www.bioconductor.org/packages/release/bioc/html/QuaternaryProd.html
ClusterProfiler	Wu et al., 2021[66]	RRID:SCR_016884
ReactomePA	Yu & He, 2016[67]	RRID:SCR_019316
ggplot2	Wickham, 2016[68]	RRID:SCR_014601
GraphPad Prism 9.3.1	GraphPad	RRID:SCR_002798

RESOURCE AVAILABILITY

Lead contact

Further information and requests for resources and reagents should be directed to and will be fulfilled by the lead contact, Ralf P. Brandes (Brandes@vrc.uni-frankfurt.de).

Materials availability

Plasmids generated in this study are available from the lead contact.

Data and code availability

- RNA-seq data have been deposited at NCBI GEO datasets and are publicly available as of the date of publication at GEO: GSE199091.
- Mass spectrometry data have been deposited under ProteomeXchange Consortium (http://proteomecentral.proteomexchange.org) via the PRIDE partner repository with the dataset identifier and are publicly available as of the date of publication at PRIDE: PXD032669.
- This paper analyzes existing, publicly available data: *PCAT19* expression across organs was analyzed using the GTEx database[1] (GTEx Analysis Release V8 (dbGaP Accession phs000424.v8.p2). FANTOM5 CAGE expression data was obtained from the FANTOM5 website (gencode v19).[2,3,4] Prostate tissue scRNA-seq data was obtained from GEO: GSE172357.[5] Haemangioma RNA-seq data was obtained from.[6] Lung endothelial scRNA-seq data was obtained from ArrayExpress: E-MTAB-6308.[7] The GEPIA database was used to analyze *PCAT19* expression between normal and cancerous tissues.[8] Tabula Sapiens data was used for gene expression analysis.[9].
- This paper does not report original code.
- Any additional information required to reanalyze the data reported in this paper is available from the lead contact upon request.

EXPERIMENTAL MODEL AND SUBJECT DETAILS

Primary cell cultures and cell lines

Pooled human umbilical vein endothelial cells (HUVEC, purchased from PromoCell, #C-12203), human microvascular endothelial cells (HMEC, from CDC, 98,247, male), human coronary artery endothelial cells (HCAEC, from PeloBiotech, PB-CH-182-2011, QC06814F10) and human aortic endothelial cells (HAoEC, purchased from PeloBiotech, 304K-05a, Lot No. 2366, male) were cultured on gelatine-coated plates in endothelial growth medium (EGM) containing 12% (for HUVEC, HMEC, HCAEC) or 20% (for HAoEC) fetal calf serum (FCS, S0113, Biochrom, Germany), penicillin (50 U/mL) and streptomycin (50 μg/mL) (15,140-122, Gibco/Lifetechnologies, USA) in a humidified atmosphere of 5% CO_2 at 37°C. The different batches of HUVEC were all commercial pools of cells obtained from umbilical cord/umbilical vein of caucasians (474Z010: 2 males, 1 female; 408Z014: 2 males, 1 female; 471Z011: 2 males, 2 females; 466Z022: 2

males, 1 female). HUVEC that had been frozen and stored at passage two, were seeded for passage three and used for experiments after seeding to passage four. The seeding density was dependent on the experiment to be performed. Standard seeding conditions (50,000 cells/cm^2) were used for experiments such as protein or chromatin immunoprecipitation. Experiments involving RNA interference required a cell seeding density of 25,000 cells/cm^2 for next day transfection. Cell cycle-related experiments also required a low seeding density to ensure continued cycling. For each experiment, at least three different batches of HUVEC from passage 3 were used.

Human dermal lymphatic endothelial cells (HDLEC, C-12217; Lot No. 394Z027.3, 4092401.3, both female) were purchased from Promocell (Heidelberg, Germany) and cultured in a humidified atmosphere of 5% CO_2 at 37°C in endothelial cell growth medium MV2 (Promocell, Heidelberg, Germany). HEK-293 (293, ATCC, CRL-1573) and HEK293T (293T/17 [HEK 293T/17], ATCC, CRL-11268) cells were cultured in DMEM (Gibco) supplemented with 10% fetal bovine serum (FBS) and 1% Pen-Strep in a humidified atmosphere of 5% CO_2 at 37°C.

Human induced pluripotent stem cells (hiPSCs, WSTIi081-A, EbiSC, male) were used for the generation of cardiac organoids. In brief, 500 hiPSCs were cultured for 2 d on ultra-low-attachment surface in TeSR™-E8™ medium (#05990, STEMCELL™ Technologies) at 37°C and 5% CO_2 in a humidified atmosphere to form iPSC-aggregates.

METHOD DETAILS

Cell stimulations

HUVEC were seeded the day before stimulation and cultured as described above. The following chemicals were used in cell stimulation experiments: Human recombinant VEGF-A 165 (50 and 100 ng/mL; R&D, 293-VE), camptothecin (1 μM and 10 μM), ATR inhibitor (10 μM, VE-821, Selleckchem) and hydroxyurea (2mM, Sigma-Aldrich). Stimulations were performed in either EGM (12% FCS) or in EBM (6% FCS) (e.g. spheroid VEGF-A stimulations). The duration of stimulations varied between experiments and is therefore indicated in the individual figure legends.

LNA GapmeR-mediated knockdown

Cells were seeded at a density of 25,000 cells/cm^2 one day before transfection with LNA GapmeRs (Qiagen). Cells were transfected with LNAs using the RNAiMAX transfection reagent according to the manufacturer's protocol (Qiagen). A final LNA concentration of 30 nM was used for 48–72h before stopping cells with either RNA lysis buffer or protein lysis buffer. In some cases, cells were reseeded for further experiments. LNA GapmeRs were designed with the Qiagen/Exiqon LNA probe designer and had the following sequences: *PCAT19* 5′-AATTCGGCTCTTACAA-3′ and as a negative Control 5′-AACACGTCTATACGC-3′.

Overexpression

700,000 cells were resuspended and electroporated in E2 buffer with the NEON electroporation system (Invitrogen) (1,400 V, 1 × 30 ms pulse). 7 μg of plasmid was used for each overexpression. A full medium exchange was performed every 24 h and cells were incubated for a total of 48 h. The following plasmids were used: pcDNA3.1 + vector containing *PCAT19* and pcDNA3.1 + as a negative control.

EdU proliferation assay

Cells were seeded at a density of 10,000 cells/cm^2 in ibidi 8-well plates. After 24 h a 2X working solution of EdU (C10337, ThermoFisher) in EGM was added to the cells for 6 h. 4% paraformaldehyde (PFA) was added to the cell medium for 15 min before washing with 3% BSA in PBS and then 0.5% Triton X- for 20 min. Cells were washed again with 3% BSA before the addition of a Click-iT reaction cocktail (Click-iT reaction buffer, $CuSO_4$ (Component E), Alexa Fluor Azid and Click-iT buffer additive) for 30 min at RT. Cells were washed and incubated in Hoechst 33,342 (Component G) solution 1:2000 in PBS (5 μg/mL) for a further 30 min at RT before washing with PBS. Cells were imaged for Hoechst and EdU (488 nm) with a laser scanning confocal microscope (LSM800, Zeiss) and images quantified with FIJI/ImageJ.[56]

Spheroid outgrowth assay

HUVEC spheroid outgrowth assays were performed as described previously.[69] Spheroids were stimulated in EBM (6% FCS) containing 50 ng/mL VEGF-A 165 for 16 h before the addition of 4% PFA to the medium. Images of 10 spheroids per condition and replicate were acquired using an Evos XL Core microscope (Life technologies) and outgrowth length and numbers quantified using ImageJ.

Human cardiac organoid formation

iPSC-aggregates were differentiated to cardiac organoids (hCOs) using the STEMdiff Cardiomyocyte Differentiation Kit (#05010, STEMCELL Technologies) following the instructions from the supplier. hCOs were then maintained in mixed medium of STEMdiff Cardiomyocyte Maintenance Basal Medium (#05020, STEMCELL Technologies) and Endothelial Cell Growth Medium 2 (#C-22111, PromoCell) at a ratio of 4:1, with medium changes every second day for a further 28 d. Medium was then changed to medium supplemented with 140 nM CTL LNA or *PCAT19* LNA for 48 h. hCOs were then fixed with 4% PFA overnight at 4°C. Whole mount staining was then performed by incubating hCOs in 1% Triton X-100 for 1 h, followed by blocking in 5% horse serum for 1 h.

hCOs were immunostained with primary antibody solution (1:200 anti-alpha actinin (#A7811, Sigma Aldrich), 1:200 anti-VE-Cadherin (#2500, Cell Signaling Technologies)) at 4°C overnight and secondary antibody solution (1:500 anti-mouse AlexaFlour488 (Invitrogen, A11017) and 1:500 anti-rabbit Alexa Fluor 647 (Invitrogen, A21246)) at RT for 3 h, followed by 2 h of washing in 1X PBST. Nuclei were counterstained with DAPI. The stained hCOs were transferred onto glass slides and imaged with the Leica SP8 Confocal System. The whole hCO was imaged using a z stack between two ends of the organoid. Organoids were quantified for cumulative vascular network length and organoid diameter using the Leica LAS X software.

DNA fiber assay

HUVEC were sequentially labeled with 5-Chloro-2′-deoxyuridine (CldU, 50 μM) and 5-Iodo-2′-deoxyuridine (IdU, 50 μM) for 15 min. After labeling, cells were trypsinised, resuspended in cold PBS, diluted to 1.75×10^5/mL and mixed 1:1 with unlabelled cells. 7.5 μL lysis buffer (200 mM Tris-HCl pH 7.5, 50 mM EDTA, 0.5% SDS) was mixed with 4 μL of the cell suspension on a SuperFrost Plus microscopy slide (ThermoFisher), incubated horizontally for 9 min and tilted, allowing the solution to spread to the bottom of the slide. Following air-drying, DNA spreads were fixed with 3:1 methanol:acetic acid overnight at 4°C. The spreads were then rehydrated 3 × 3 min in PBS, denatured in 2.5 M HCl for 1.5 h at RT, then washed 5 × 2.5 min in PBS. The slides were blocked for 40 min in blocking solution (2% BSA in PBS-T), followed by incubation with primary antibodies (mouse anti-BrdU, 1:100, BD Bioscience and rat anti-BrdU, 1:100, Abcam) at RT for 2.5 h. After 3 × 5 min washes with PBS-T, the slides were incubated with secondary antibodies (goat anti-mouse Alexa Fluor 647, 1:500, Thermo Scientific and goat anti-rat Alexa Fluor 488, 1:500, Thermo Scientific) at RT for 1 h. The slides were then washed 3 × 5 min with PBS-T, air-dried and mounted with Prolong Gold AntiFade Mountant (Thermo Scientific). Images of DNA fibers were acquired with a Widefield Fluorescence Microscope (Thunder, LASX software, Leica) (magnification: 100x, NA 1.44 HC PL APO oil immersion objective; LED illumination and the corresponding emission filters: 635 nm, 642/80 and 475 nm, 535/70). Lengths of DNA fibers were quantified using the Fiji/ImageJ software.

Overexpression and purification of RPA2 proteins

Recombinant overexpression of full-length RPA2 protein was achieved using Turbo E.coli chemically competent cells (NEB, catalog number: C2984H). Recombinant plasmid (pVM_MBP) was transformed by heat-shock on Luria broth agar plates and colonies were inoculated the next day in fresh Luria broth medium supplemented with 100 μg/mL ampicillin and cultured overnight at 37°C. Overexpression was induced at OD600 = 0.7 using a final concentration of 0.4 mM isopropyl-β-D-1-thiogalactopyranoside (IPTG) and the cultures were further left to grow at 18°C overnight. Cells were harvested the next day by centrifugation and lysed by sonication in lysis buffer (50 mM Tris pH 7.5, 500 mM NaCl, 5% (v/v) Glycerol, 15 mM imidazole) supplemented with an EDTA-free protease inhibitor cocktail tablet (Roche Applied Science) and 30 μg/mL DNase I. The lysate was cleared by centrifuging at 10,000 rpm for 1 h and filtered using a 0.22 μM filter membrane, before applying the lysate to Nickel-NTA metal affinity agarose resin beads (Cube Biotech) pre-equilibrated in lysis buffer. The lysate was left to incubate on the beads at 4°C for 1 h, and the flow-through was removed after gentle centrifugation at 300 g for 2 min. The beads were washed 5 times with column volume of lysis buffer and an incubation time was 10 min at 4°C with subsequent gentle centrifugation during each step. Elutions were performed using lysis buffer supplemented with increasing imidazole concentrations of 50 mM, 100 mM, and 200 mM and 500 mM and an incubation time of 10 min and subsequent gentle centrifugation. The purest fractions, determined by SDS-PAGE, were concentrated using Merck-Millipore centricons by centrifugation at 4,500 rpm and loaded onto a HiLoad Superdex S200 10/300 GL (GE Healthcare) column previously equilibrated in gel filtration buffer (25 mM Tris pH 7.5, 200 mM NaCl, 0.5 mM TCEP) for size exclusion chromatography. Concentrated proteins were used later for all *in vitro* or semi-*in-vitro* interaction studies.

In vitro transcription and RNA 3′end biotinylation

pcDNA3.1+*PCAT19* or control pcDNA3.1+ plasmid DNA were linearised with SmaI (ThermoFisher) and purified. DNA was *in vitro* transcribed according to the manufacturer's protocol with T7 Phage RNA Polymerase (NEB). Afterward, the remaining DNA was digested with RQ DNase I (Promega). The *in vitro* transcribed RNA was purified with the RNeasy Mini Kit (Qiagen) and biotinylated at the 3′end with the Pierce RNA 3′end biotinylation kit (ThermoFisher).

PCAT19-RPA2 *in vitro* assays

For the *in vitro* interaction assay, purified recombinant RPA2 protein (5 μg) was mixed with *in vitro*-transcribed Biotin-*PCAT19* (300 ng) in a reaction containing 1 μL/mL (20 units/mL) SUPERaseIN inhibitor for 2 h at RT. For the *in vitro* phosphorylation assay, purified RPA2 protein (10 μg), *in vitro* transcribed Biotin-*PCAT19* (300 ng) and HUVEC crude cell lysate (200 μg) were mixed in kinase reaction buffer (20 mM HEPES (pH 7.5), 10 mM $MgCl_2$, 1 mM dithiothreitol, and 0.3 μm ATP) containing 1 μL/mL (20 units/mL) SUPERaseIN inhibitor (ThermoFisher) and ssDNA from salmon sperm (100 μg/mL) (ThermoFisher). The mixture was incubated for 30 min at 37°C and alternatively, 20 μM ATR inhibitor (VE-821) (Selleckchem) or phosphatase (100 U/mL) (Merck) were added to the mixture before incubation. Importantly, biotin-*PCAT19* RNA or biotin-pcDNA3.1+ control RNA was previously folded and added to the respective mixtures (*in vitro* phosphorylation assay and *in vitro* protein interaction experiment) at equimolar concentrations. Lastly, biotinylated labeled substrates were captured with 20 μL Streptavidin Magnetic Beads (NEB) and incubating the mixture overnight at 4°C. Beads were washed 4 times with cold PBS-T (0.1% Tween 20) and then boiled in 20 μL 1x Laemmli SDS sample buffer (ThermoFisher) for

10 min. Samples were applied to SDS-PAGE and Western Blotting and the detection of biotinylated-proteins was performed with the Odyssey CLx Imaging System.

For the semi-*in vitro* phosphorylation assay in HEK293T lysate, purified RPA2 protein (10 μg), *in vitro* transcribed Biotin-*PCAT19* (300 ng) and HEK293T crude cell lysate (200 μg) (of transfected cells the day before with 10 μg CMV Flag ATRwt (gift from Stephen Elledge (Addgene plasmid #41909; http://n2t.net/addgene:41909; RRID:Addgene_41909)[53] with PEI (Polyethylenimine, linear, MW 25000, Polysciences, Cat# 23966)) were mixed in kinase reaction buffer (20 mM HEPES (pH 7.5), 10 mM $MgCl_2$, 1 mM dithiothreitol, and 0.3 μm ATP) containing 1 μL/mL (20 units/mL) SUPERaseIN inhibitor (ThermoFisher) and ssDNA from salmon sperm (100 μg/mL) (ThermoFisher). The mixture was incubated for 30 min at 37°C and alternatively, 20 μM ATR inhibitor (VE-821) (Selleckchem) or phosphatase (100 U/mL) (Merck) were added to the mixture before incubation. Samples were applied to SDS-PAGE and Western Blotting and the detection of biotinylated-proteins was performed with the Odyssey CLx Imaging System.

RNA isolation, reverse transcription and RT-qPCR

Total RNA was isolated and purified from HUVEC using the RNA Mini Kit according to the manufacturer's protocol (Bio&SELL). Purified RNA was reverse transcribed with SuperScript III Reverse Transcriptase (Thermo Fisher) and oligo(dT)23 together with random hexamer primers (Sigma). cDNA was quantified with RT-qPCR using ITaq Universal SYBR Green Supermix with ROX as reference dye (Bio-Rad, 1,725,125) in an AriaMX cycler (Agilent). Human target genes were normalised to GAPDH. Relative expressions were calculated using the ΔΔCt method with the AriaMX qPCR software (Agilent). Primers used in this study are listed in Table S3.

Protein isolation and western blot by SDS-PAGE

HUVECs washed in Hanks solution (Applichem) were lysed with buffer A (10 mM HEPES pH 7.9, 10 mM KCl, 0.1 mM EDTA, 0.1 mM EGTA, protein inhibitor mix (PIM), Phenylmethylsulfonylfluoride (PMSF), and DTT). After 10 min incubation at 4°C, 0.75% nonidet was added to the lysate, vortexed for 10 s and centrifuged for 1 min at 16,000 g. Nuclear pellets were resuspended in buffer C (20 mM HEPES pH 7.9, 0.4 mM NaCl, 1 mM EDTA, 1 mM EGTA, protein inhibitor mix (PIM), Phenylmethylsulfonylfluoride (PMSF), and DTT) for 15 min at 4°C before centrifugation for 1 min, 16,000 g. Protein concentrations of the supernatant were determined with the Bradford assay and the cell extract was boiled in Laemmli buffer. Equal amounts of protein were separated with SDS-PAGE and the gels were blotted onto a nitrocellulose membrane and blocked in Rotiblock (Carl Roth, Germany). After incubation with the first antibody, infrared-fluorescent-dye-conjugated secondary antibodies (Licor, Bad Homburg, Germany) were used and signals detected with an infrared-based laser scanning detection system (Odyssey Classic, Licor, Bad Homburg, Germany). Images were acquired with the Image Studio Ver 5.2 software (Licor). The following antibodies were used: RPA2 (ab2175, Abcam) RPA2 (A300-244A, Bethyl), S33-pRPA2 (A300-246A, Bethyl), T21-pRPA2 (ab61065, Abcam), S4/8-pRPA2 (A700-009, Bethyl), p53 (sc-6243, Santa Cruz), Lamin B1 (sc-20682, Santa Cruz), His6 (11922416001, Roche), DYKDDDDK Tag (D6W5B) (FLAG, 14793, Cell Signaling Technology), GAPDH (G8795, Sigma-Aldrich), Beta-actin (A1978, Sigma-Aldrich), PNKP (A300-257A, Bethyl), yH2AX (MABE205, Millipore), H2A (3636, Cell Signaling).

RNA immunoprecipitation

3×10^{15} HUVEC were grown to 80% confluence and washed once with Hanks buffer. 6 mL Hanks buffer was added to the cells on ice and irradiated with 0.150 J/cm2 254 nm UV light (BIO-LINK, BLX-254, Vilber). Cells were scraped twice in Hanks buffer and centrifuged at 1,000 g at 4°C for 4 min. Isolation and lysis of the nucleus was performed as outlined above for protein isolation and immunoprecipitation. 10% of the nuclear lysate served as the "input". 4 μg anti-RPA2 (A300-244A, Bethyl) or anti-IgG (ab37415, Abcam) negative control antibody were pre-coupled to 50 μL protein A magnetic beads (ThermoFisher) in buffer C for 1 h at RT then washed once with high salt buffer (1 M NaCl) and twice with buffer C3. The antibody-coupled beads were added to the nuclear lysate and rotated for 1 h at 4°C. Samples were placed on a magnetic bar and the lysate discarded. The beads were washed three times in high salt buffer (4°C for 10 min). Beads were then washed twice in buffer PNK (350 mM Tris-HCl pH 6.5, 50 mM $MgCl_2$, 5 mM DTT). For elution of RNA, all PNK buffer was removed and RNA isolation performed with QIAzol (Qiagen) according to the manufacturer's protocol.

Antisense-oligonucleotide pulldown of RNA

Antisense oligonucleotides containing a 5′-biotin tag were designed with the online GeneGlobe tool (QIAgen) using the target RNA sequence as input. HUVEC were UV-crosslinked on ice (0.150 J/cm2 254 nm UV light (BIO-LINK, BLX-254, Vilber)) and scraped. Cell pellets were flash frozen and thawed to disrupt the nuclei. Cells were resuspended in 200 μL buffer L (50 mM Tris/HCl pH8, 50 mM NaCl, 0.5% NP-40, 1 mM EDTA, protein inhibitor mix (PIM), Phenylmethylsulfonylfluoride (PMSF), DTT and superase 1μL/mL), incubated on ice for 30 min and centrifuged at 10,000 g at 4°C for 3 min 1mL buffer L and 20 μL MyOne Streptavidin C1 beads were added to the lysate for 30 min at 4°C. The beads were discarded and 200 pmol of *PCAT19* antisense-oligonucleotide (5′-AAGCAGACATGAGACCTCACT-3′) or scramble control oligonucleotide (5′-GTGTAACACGTCTATACGCCCA-3′) added to the pre-cleared lysate for rotation overnight at 4°C. The next day, 50 μL MyOne Streptavidin C1 beads were added to the samples for rotation at 4°C for 2 h. Beads were then washed and used for mass spectrometry or cooked in Laemmli buffer for Western blotting as described above.

CRISPR/dCas9 activation (CRISPRa) and inactivation (CRISPRi)

Guide RNAs (gRNA) were designed with the help of the web-interfaces of CRISPick GPP sgRNA designer.[57] For CRISPRa, a catalytically inactive Cas9 (dCas9) fused to the transcription activator VP64 (pHAGE EF1α dCas9-VP64) was used. For CRISPRi, a dCas9 fusion to the KRAB repressive domain (pHAGE EF1α dCas9-KRAB) was used. Either of them was transfected in HUVEC together with a sgRNA(MS2) vector containing the individual guide RNA (gRNA) using the NEON electroporation system (Invitrogen). pHAGE EF1α dCas9-VP64 and pHAGE EF1α dCas9-KRAB were a gift from Rene Maehr and Scot Wolfe (Addgene plasmid # 50918, # 50919)[54] and sgRNA(MS2) cloning backbone was a gift from Feng Zhang (Addgene plasmid # 61424).[55] The following oligonucleotides were used for cloning of the guide RNAs into the sgRNA(MS2) vector: For CRISPRa, 5′-CACCGAATGTGCAGGACTCATCAAC-3′ and 5′-AAACGTTGATGAGTCCTGCACATTC-3′, and for CRISPRi 5′-CACCGAGTGTTATTTGACTGGAGTG-3′ and 5′-AAACCACTCCAGTCAAATAACACTC-3′. After cloning, plasmids were purified and sequenced.

Mass spectrometry

Immunoprecipitation was performed as above but with the final wash of IP beads in wash buffer without protease inhibitors. Beads were transferred to fresh low-binding tubes in order not to disrupt protein digestion and to remove sticky proteins. Beads were flash frozen in liquid nitrogen and subjected to mass spectrometry. Briefly, samples underwent digestion with trypsin (Promega, Walldorf, Germany) overnight at 37°C and stopped with trifluoroacetic acid (Sigma-Aldrich). Peptides were purified with multi-stop-and-go tips (StageTips).[70] Liquid chromatography/mass spectrometry (LC/MS) was performed on Thermo Scientific Q Exactive Plus equipped with an ultra-high performance liquid chromatography unit (Thermo Scientific Dionex Ultimate 3000) and a Nanospray Flex Ion-Source (Thermo Scientific). Peptides were loaded and separated using gradient phases. MaxQuant 1.6.1.0132[58] and Perseus 1.6.1.3[59] were used for data analysis. The human reference proteome set (Uniprot) was used to identify peptides and proteins with a false discovery rate (FDR) of less than 1%. Reverse identifications and common contaminants were removed and the dataset was reduced to proteins that were identified in at least 4 of 6 samples in one experimental group. Missing LFQ values were replaced by random background values. Significant interacting proteins were determined by permutation-based false discovery rate (FDR) calculation and students t-test. The abundance of each protein was determined using the iBAQ value, which is measured by dividing the sum of peptide intensities the number of theoretically observable peptides.[71]

A detailed description and the mass spectrometry proteomics data have been deposited to the ProteomeXchange Consortium (http://proteomecentral.proteomexchange.org) via the PRIDE partner repository with the dataset identifier PRIDE: PXD032669.

Proximity ligation assay (PLA)

The PLA was performed similarly as described in the manufacturer's protocol (Duolink II Fluorescence, OLink, Upsalla, Sweden). After fixation in phosphate buffered formaldehyde solution (4%), HUVEC were permeabilized with Triton X-100 (0.2%) and blocked with serum albumin solution (3%) in phosphate-buffered saline. After incubation overnight with anti-RPA2 (A300-244A, Bethyl) and anti-ATR (sc-515173, Santa Cruz), samples were washed and incubated with the respective PLA-probes for 1 h at 37°C. After washing and ligation for 30 min (37°C), the amplification with polymerase was performed for 100 min (37°C). The nuclei were stained using DAPI. Images (with Alexa Fluor, 546 nm) were acquired with a confocal microscope (LSM 800, Zeiss) and the number of PLA signals was normalised to the number of nuclei per image.

RNA-fluorescent *in situ* hybridisation (FISH)

RNA-FISH was performed to determine the subcellular localisation of RNAs of interest. Cells that had been grown on 8-well culture plates (Ibidi) were fixed in 4% PFA for 7 min at RT and washed 3 times with PBS. Cells were permeabilised in 0.5% Triton X-100 containing 1 μL/mL SuperaseIN on ice for 10 min. Cells were washed three times in PBS for 5 min each and rinsed with 2XSSC buffer. Hybridisation was then performed overnight at 37°C in hybridisation buffer containing 100 μM antisense oligonucleotide probes with a 5′-TYE tag. *PCAT19* antisense-oligonucleotide (5′-AAGCAGACATGAGACCTCACT-3′) or scramble control oligonucleotide (5′-GTGTAACACGTCTATACGCCCA-3′). The next day, cells were washed four times for 20 min each in 2XSSC buffer containing 50% formamide at 37°C. DAPI staining (1:200) was included in the second wash step. Cells were imaged with a laser scanning confocal microscope (LSM800) and images quantified with FIJI/ImageJ.

RNA *in situ* hybridization-proximity ligation assay (rISH-PLA)

10,000 HUVECs were grown on 8-well ibidi slides, treated as indicated, and were fixed using 4% paraformaldehyde for seven minutes. To confirm the interaction between *PCAT19* and γH2AX, the rISH-PLA assay was performed as described elsewhere[72] with the biotinylated *PCAT19* oligonucleotide, an anti-biotin antibody (Anti-Biotin antibody [Hyb-8] (ab201341, Abcam)) and an anti-γH2AX antibody (MABE205, Millipore).

Terminal deoxynucleotidyl transferase dUTP nick end labeling (TUNEL)

The TUNEL assay was used to detect single- and double-stranded DNA breaks according to the manufacturer's protocol (TMR red, Sigma-Aldrich). Briefly, cells that had been grown on 8-well culture plates (Ibidi) were fixed in 4% PFA for 1 h at RT. Cells were rinsed with PBS and incubated in 0.1% TritionX-100 containing 0.1% sodium citrate for 2 min on ice. Cells were then incubated in 1:2 TUNEL

reaction mixture for 60 min at 37°C in the dark. Cells were rinsed 3 times with PBS and DAPI staining (1:200) included in the second wash. Cells were imaged with a laser scanning confocal microscope (LSM800) and images quantified with FIJI/ImageJ.

Comet assay

The comet assay was used to detect DNA damage by single-cell gel electrophoresis according to the manufacturer's protocol (CometAssay Single Cell Gel Electrophoresis Assay, 4250-050-K, R&D Systems). Briefly, cells were treated with and without DNA damaging agents (as indicated in figure legends). Cells treated with 100 μM H_2O_2 for 20 min at 4°C served as a positive control. Cells were trypsinised, counted and 1×10^5 cells mixed with low-melting agarose before being placed on prewarmed comet slides. Slides were stored in the dark at 4°C for 30 min then immersed in lysis solution for 60 min at RT. Slides were then immersed in alkaline unwinding solution for 20 min at RT. Slides were placed in an electrophoresis chamber and 21 V applied for 30 min before immersing slides twice in distilled H_2O for 5 min, then 70% ethanol for 5 min. Slides were dried for 15 min at 37°C and 100 μL SYBR added to the cells for 30 min at RT. Slides were then briefly rinsed in distilled H_2O and dried completely at 37°C. Cells were imaged with a laser scanning confocal microscope (LSM800) and images quantified with CometScore 2.0 (TriTek Corp).

BrdU/PI FACS

Cells were grown on 6cm culture plates and incubated with 10μM BrdU (10280879001, Roche) for 30 min before washing in 3% BSA and centrifugation at 500 g for 10 min. Cells were resuspended in 70% ethanol while vortexing and then incubated on ice for 30 min. Cells were centrifuged again at 500 g for 10 min and resuspended in 2 mM HCl containing 0.5% Triton X-100 for 30 min at RT. Cells were then resuspended in 0.1 M $Na_2B_4O_7$ for 2 min. Cells were centrifuged again and resuspended in PBS/BSA +0.05% Tween 20 with 1:100 antibody (rat anti-BrdU (ab6326, Abcam)) overnight at 4°C. Cells were then incubated with 1:500 secondary antibody (anti-rat 488nm) for 30 min at 4°C, before washing and staining with 10 μg/mL Propidium Iodide in 1% BSA containing 20 μg/mL RNase (00552782, ThermoFisher) for 20 min at 4°C. Cells were then resuspended in 1% BSA containing containing 10 μg/mL Propidium Iodide for FACS analysis. Cells were subjected to FACS analysis (SH800, Sony) using the FL2 (500–550 nm) and FL3 (570–630 nm) filters for BrdU and propidium iodide detection. Data was analyzed using the FlowJo^{TM} v10.8 Software (BD Life Sciences).

RNA-sequencing

RNA-sequencing was performed as described previously.[73] Briefly, total RNA and library integrity were verified and 600 ng of total RNA used as input for SMARTer Stranded Total RNA Sample Prep Kit - HI Mammalian (Takara Bio). Sequencing was performed on a NextSeq2000 instrument (Illumina) using a P2 flowcell with v3 chemistry, resulting in an average of 36M reads per library with 1 × 72bp single end setup. The resulting raw reads were assessed for quality, adapter content and duplication rates with FastQC.[60] Trimmomatic version 0.39[61] was employed to trim reads after a quality drop below a mean of Q20 in a window of 10 nucleotides. Only reads between 30 and 150 nucleotides were cleared for further analyses. Trimmed and filtered reads were aligned to the Ensembl human genome version hg38 (ensembl release 104) using STAR 2.74.9a[62] with the parameter "–outFilterMismatchNoverLmax 0.1" to increase the maximum ratio of mismatches to mapped length to 10%. The number of reads aligning to genes was counted with featureCounts 2.0.2[63] tool from the Subread package. Only reads mapping at least partially inside exons were admitted and aggregated per gene. Reads overlapping multiple genes or aligning to multiple regions were excluded. Differentially expressed genes were identified using DESeq2 version 1.30.1.[64] Further analysis of RNA-seq data was performed with QuaternaryProd,[65] ClusterProfiler[66] and ReactomePA[67] and visualised with ggplot2.[68]

Human carotid artery plaques

Human carotid artery plaque specimens were harvested during carotid endarterectomies (CEA) performed in the Department for Vascular and Endovascular Surgery at the Klinikum rechts der Isar of the Technical University Munich. The study was approved by the local Ethics Committee, and all patients provided their written informed consent in accordance with the Declaration of Helsinki. Two types of analysis were performed as described previously: stable (n = 6) vs. unstable (n = 5) plaques[74] based on the Rothwell/Redgrave criteria[75] (fibrous caps >200μm are considered stable, fibrous caps <200μm are rendered unstable or ruptured); as well as late stage, advanced atherosclerotic plaques (n = 12) compared to early diseased/healthy control (n = 10) specimens stemming from the same individual.[76] Plaque samples underwent basic stains to assess and characterise plaque morphology using hematoxylin & eosin (HE) as well as Elastica van Giesson (EvG) protocols. For molecular analysis, plaques were placed in RNA later (Qiagen) for 24h, before being frozen at −80°C for further analysis. Both of the plaques settings were sent for bulk RNA-sequencing, as described previously.[74,76]

QUANTIFICATION AND STATISTICAL ANALYSIS

Results are presented as mean ± standard deviation (SD). Statistical significance was calculated using GraphPad Prism 9.3.1. For multiple comparisons testing One-way ANOVA with Tukey multiple comparisons test was employed. The students t-test (paired or unpaired) was performed for experiments where only two conditions were included. Statistical analysis for RNA-sequencing experiments were performed with the DESeq2 and Diffbind packages respectively. p-value and number of replicates (n) are displayed with each result.

Cell Reports, Volume *41*

Supplemental information

Long non-coding RNA *PCAT19* safeguards DNA in quiescent endothelial cells by preventing uncontrolled phosphorylation of RPA2

James A. Oo, Katalin Pálfi, Timothy Warwick, Ilka Wittig, Cristian Prieto-Garcia, Vigor Matkovic, Ines Tomašković, Frederike Boos, Judit Izquierdo Ponce, Tom Teichmann, Kirill Petriukov, Shaza Haydar, Lars Maegdefessel, Zhiyuan Wu, Minh Duc Pham, Jaya Krishnan, Andrew H. Baker, Stefan Günther, Helle D. Ulrich, Ivan Dikic, Matthias S. Leisegang, and Ralf P. Brandes

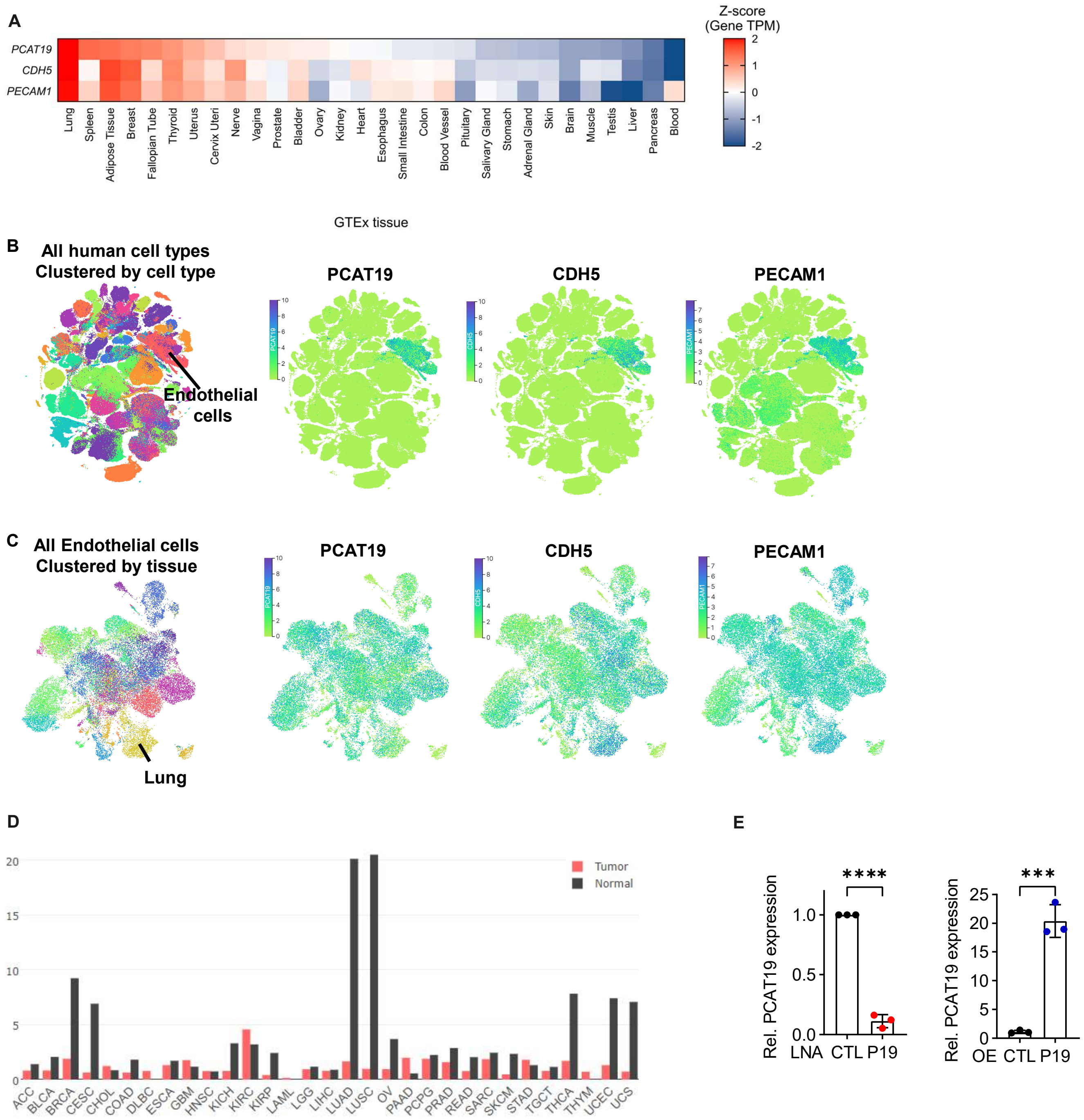

Figure S1. PCAT19 expression, Related to Figures 1 and 2. A. *PCAT19, CDH5* and *PECAM1* expression (Z-score of gene TPM) in normal human tissues from The GTEx Portal (GTEx Analysis Release V8 (dbGaP Accession phs000424.v8.p2). TPM, transcripts per million. **B.** *PCAT19*, *CDH5* and *PECAM1* expression in different human cell types. Clustered by cell type. Tabula Sapiens. **C.** *PCAT19*, *CDH5* and *PECAM1* expression in individual endothelial cells across all human tissues. Clustered by tissue. Tabula Sapiens. **D.** *PCAT19* expression in various tumour tissues and respective normal tissues, GEPIA database. **E.** RT-qPCR for PCAT19 after PCAT19 or control LNA GapmeR-mediated knockdown and PCAT19 or pcDNA3.1+ control overexpression. HUVEC (n=3 biological replicates) for both. Unpaired t-test, *** signifies $P<0.001$, **** signifies $P<0.0001$.

A

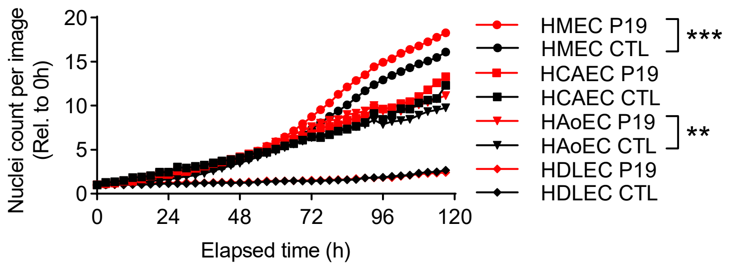

B

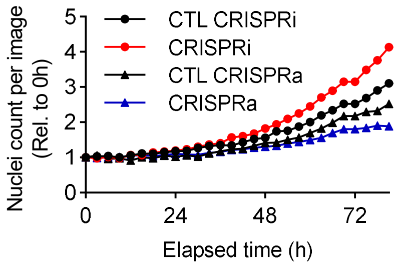

C

ID	Description	GeneRatio	BgRatio	pvalue	p.adjust	Count	FoldEnrichment
hsa04110	Cell cycle	10/78	126/8114	2.9351E-07	4.8135E-05	10	8.25600326
hsa04218	Cellular senescence	9/78	156/8114	1.6739E-05	0.00137261	9	6.00147929
hsa04914	Progesterone-mediated oocyte maturation	7/78	102/8114	5.1302E-05	0.00280453	7	7.13901458
hsa05166	Human T-cell leukemia virus 1 infection	9/78	222/8114	0.00025833	0.01059171	9	4.21725572
hsa04114	Oocyte meiosis	6/78	131/8114	0.0015673	0.05140744	6	4.76453318
hsa04010	MAPK signaling pathway	9/78	294/8114	0.001933	0.05283537	9	3.1844584
hsa05202	Transcriptional misregulation in cancer	7/78	193/8114	0.00245286	0.05746707	7	3.77295071
hsa04115	p53 signaling pathway	4/78	73/8114	0.00520871	0.10677856	4	5.70003512
hsa04657	IL-17 signaling pathway	4/78	94/8114	0.01254742	0.20036465	4	4.42662302
hsa05169	Epstein-Barr virus infection	6/78	202/8114	0.01284993	0.20036465	6	3.08987053
hsa05203	Viral carcinogenesis	6/78	204/8114	0.01343909	0.20036465	6	3.05957768
hsa04933	AGE-RAGE signaling pathway in diabetic complications	4/78	100/8114	0.01545506	0.21121909	4	4.16102564
hsa05161	Hepatitis B	5/78	162/8114	0.01955799	0.24563435	5	3.21066793
hsa04668	TNF signaling pathway	4/78	112/8114	0.02246656	0.24563435	4	3.71520147
hsa05145	Toxoplasmosis	4/78	112/8114	0.02246656	0.24563435	4	3.71520147
hsa04935	Growth hormone synthesis, secretion and action	4/78	119/8114	0.02732572	0.28008868	4	3.4966602
hsa04917	Prolactin signaling pathway	3/78	70/8114	0.02952985	0.28487624	3	4.45824176
hsa04380	Osteoclast differentiation	4/78	128/8114	0.03443394	0.29341409	4	3.25080128
hsa04926	Relaxin signaling pathway	4/78	129/8114	0.03528429	0.29341409	4	3.22560127
hsa04068	FoxO signaling pathway	4/78	131/8114	0.03702153	0.29341409	4	3.17635545
hsa04215	Apoptosis - multiple species	2/78	32/8114	0.03757132	0.29341409	2	6.50160256
Hsa05162	Measles	4/78	139/8114	0.04445916	0.31701313	4	2.99354363
Hsa05418	Fluid shear stress and atherosclerosis	4/78	139/8114	0.04445916	0.31701313	4	2.99354363

D

KEGG Description	P-adjusted Value	Gene count	Genes
Cell cycle	4.824335e-05	10	*BUB1, BUB1B, CCNA2, CCNB2, CCND2, CDC20, CDC25B, ESPL1, ORC1, TTK*
Cellular senescence	1.375276e-03	9	*CCNA2, CCNB2, CCND2, FOXM1, IGFBP3, MAP2K6, MAPK14, MYBL2, TGFBR2*
Progesterone-mediated oocyte maturation	2.808863e-03	7	*BUB1, CCNA2, CCNB2, CDC25B, CPEB4, KIF22, MAPK14*
Human T-cell leukemia virus 1 infection	1.061092e-02	9	*BUB1B, CCNA2, CCNB2, CCND2, CDC20, ESPL1, NFKB2, STAT5A, TGFBR2*

E

Symbol	Regulation	Significant reachable	*P*-value
AURKB	down	12	2.42E-10
PLK1	down	10	4.41E-09
CDK1	down	12	1.50E-07
SMARCA2	up	11	6.42E-05
SMARCA4	up	11	7.12E-05
RB1	down	6	7.32E-05
E2F3	down	7	0.000304
CCNB1	down	10	0.000319

Figure S2. PCAT19 on endothelial cell cycle, Related to Figure 2. A. Proliferation measured with Incucyte imaging system after LNA GapmeR-mediated knockdown of PCAT19 (P19) compared to negative control (CTL) LNA GapmeR for HMEC, HCAEC, HAoEC and HDLEC. n=2 technical replicates, unpaired t-test of Area under the curve (AUC). ** signifies $P<0.01$, *** signifies $P<0.001$. **B.** Endothelial cell proliferation measured with Incucyte imaging system after PCAT19 CRISPRi or CRISPRa or respective negative controls. n=3 biological replicates. **C.** KEGG (Kyoto Encyclopedia of Genes and Genomes) pathway enrichment list from 186 differentially regulated genes after *PCAT19* knockdown. **D.** Differentially expressed genes associated with the top significant KEGG pathways (*P*adj<0.05). **E.** Upstream regulator analysis (QuaternaryProd package). Top predicted regulators listed according to their number of significant downstream targets. ** signifies $P<0.01$, *** signifies $P<0.001$.

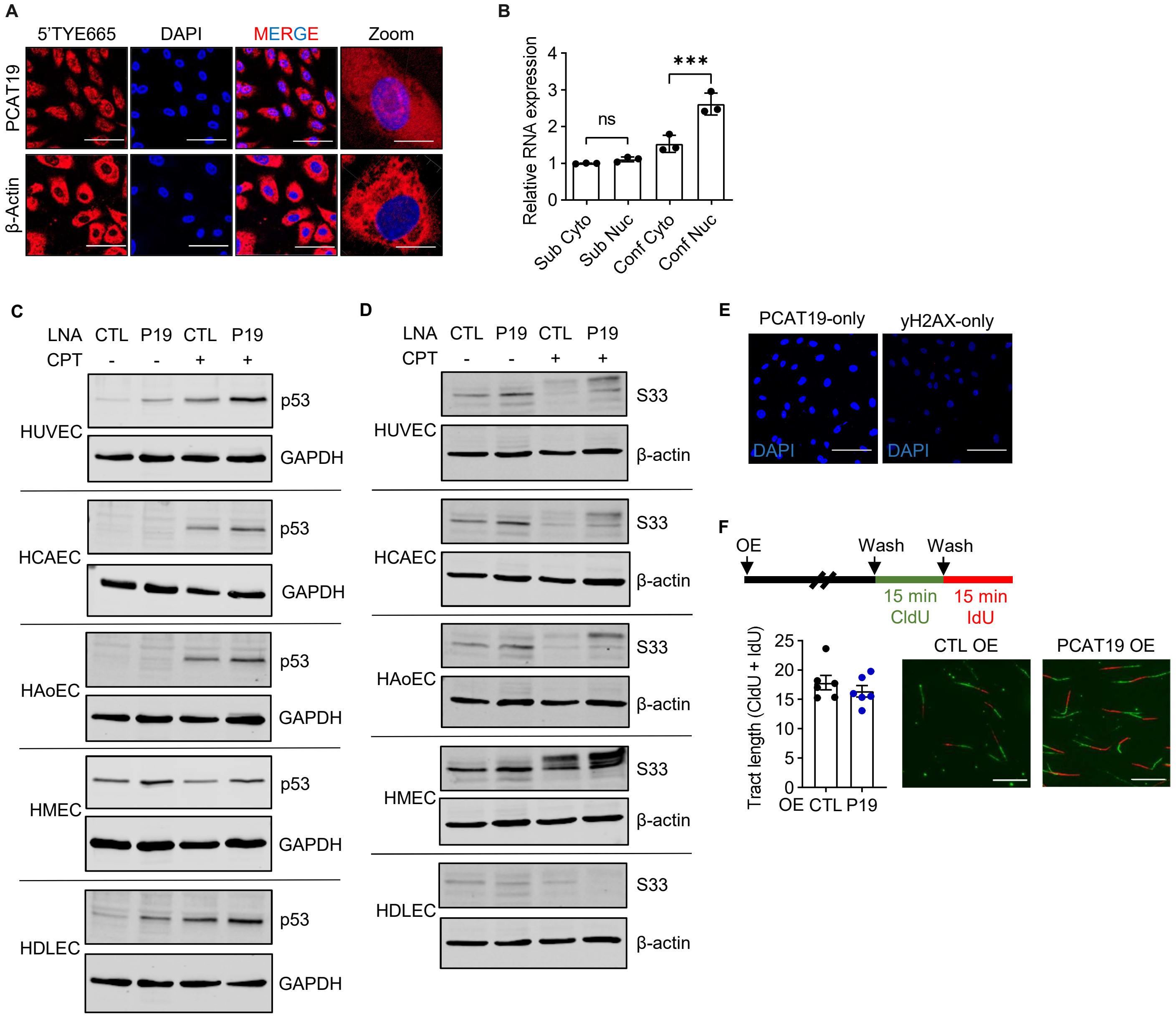

Figure S3. PCAT19 subcellular localisation and effects on DNA damage markers and DNA replication, Related to Figures 1 and 4. A. RNA-FISH for *PCAT19* and β-Actin localisation in HUVEC. Respective antisense-oligonucleotide probes tagged with 5'TYE665 (647nm). DAPI counterstaining. Scale bar represents 100 µm and "Zoom" image scale bars represent 25 µm. **B.** HUVEC fractionation into cytoplasm (Cyto) and nucleus (Nuc) after cell growth under both subconfluent (sub) and confluent (conf) conditions. RNA isolation and RT-qPCR for *PCAT19* and GAPDH. n=3, unpaired t-test, mean ± SD. **C.** HUVEC, HCAEC, HaoEC, HMEC and HDLEC were transfected with PCAT19 LNA (P19) or negative control LNA (CTL) and then treated with DMSO or camptothecin (CPT). n=1. Western blot staining for p53 and GAPDH or **D.** S33-pRPA2 (S33) and β-actin. **E.** RNA In Situ Hybridization Proximity Ligation Assay (rISH-PLA) PCAT19- and γH2AX-only controls. Red signal indicates PLA signal (546nm) between PCAT19 and γH2AX, blue indicates DAPI. Scale bar represents 100 µm **F.** HUVEC were transduced with PCAT19 or pcDNA3.1+ control plasmids and pulsed with CldU and IdU for the DNA fibre assay. Quantification of fibres and representative images are displayed. (n=6) Scale bar represents 15 µm. *** signifies *P*<0.001.

Table S3. Primers used in this study, Related to Figures 2 and 3.

Name	Sequence (5’-3’)
18S rRNA FP	CTT TGG TCG CTC GCT CCT C
18S rRNA RP	CTG ACC GGG TTG GTT TTG AT
GAPDH FP	TGC ACC ACC AAC TGC TTA GC
GAPDH RP	GGC ATG GAC TGT GGT CAT GAG
PCAT19 FP	ACC CTG CCC TTA GTC AAA TC
PCAT19 RP	TGG AAT CCC ACA CTG TTA CC
U4 snRNA FP	GCC AAT GAG GTT TAT CCG AGG
U4 snRNA RP	TCA AAA ATT GCC AAT GCC G
